# Bioactive Natural Products in Actinobacteria Isolated in Rainwater From Storm Clouds Transported by Western Winds in Spain

**DOI:** 10.3389/fmicb.2021.773095

**Published:** 2021-11-10

**Authors:** Aida Sarmiento-Vizcaíno, Jesús Martín, Fernando Reyes, Luis A. García, Gloria Blanco

**Affiliations:** ^1^Departamento de Biología Funcional, Área de Microbiología, Universidad de Oviedo, Oviedo, Spain; ^2^Instituto Universitario de Oncología del Principado de Asturias, Universidad de Oviedo, Oviedo, Spain; ^3^Instituto de Investigación Sanitaria del Principado de Asturias (ISPA), Universidad de Oviedo, Oviedo, Spain; ^4^Fundación MEDINA, Centro de Excelencia en Investigación de Medicamentos Innovadores en Andalucía, Granada, Spain; ^5^Departamento de Ingeniería Química y Tecnología del Medio Ambiente, Área de Ingeniería Química, Universidad de Oviedo, Oviedo, Spain

**Keywords:** *Streptomyces*, *Nocardiopsis*, actinomycetes, antibiotic, antimicrobial, antitumor

## Abstract

Actinobacteria are the main producers of bioactive natural products essential for human health. Although their diversity in the atmosphere remains largely unexplored, using a multidisciplinary approach, we studied here 27 antibiotic producing Actinobacteria strains, isolated from 13 different precipitation events at three locations in Northern and Southern Spain. Rain samples were collected throughout 2013–2016, from events with prevailing Western winds. NOAA HYSPLIT meteorological analyses were used to estimate the sources and trajectories of the air-mass that caused the rainfall events. Five-day backward air masses trajectories of the diverse events reveals a main oceanic source from the North Atlantic Ocean, and in some events long range transport from the Pacific and the Arctic Oceans; terrestrial sources from continental North America and Western Europe were also estimated. Different strains were isolated depending on the precipitation event and the latitude of the sampling site. Taxonomic identification by 16S rRNA sequencing and phylogenetic analysis revealed these strains to belong to two Actinobacteria genera. Most of the isolates belong to the genus *Streptomyces*, thus increasing the number of species of this genus isolated from the atmosphere. Furthermore, five strains belonging to the rare Actinobacterial genus *Nocardiopsis* were isolated in some events. These results reinforce our previous *Streptomyces* atmospheric dispersion model, which we extend herein to the genus *Nocardiopsis.* Production of bioactive secondary metabolites was analyzed by LC-UV-MS. Comparative analyses of *Streptomyces* and *Nocardiopsis* metabolites with natural product databases led to the identification of multiple, chemically diverse, compounds. Among bioactive natural products identified 55% are antibiotics, both antibacterial and antifungal, and 23% have antitumor or cytotoxic properties; also compounds with antiparasitic, anti-inflammatory, immunosuppressive, antiviral, insecticidal, neuroprotective, anti-arthritic activities were found. Our findings suggest that over time, through samples collected from different precipitation events, and space, in different sampling places, we can have access to a great diversity of Actinobacteria producing an extraordinary reservoir of bioactive natural products, from remote and very distant origins, thus highlighting the atmosphere as a contrasted source for the discovery of novel compounds of relevance in medicine and biotechnology.

## Introduction

In nature, members of the Phylum *Actinobacteria* continue to be the main producers of structurally diverse bioactive natural products, essential for human health. Among Actinobacteria, species of the *Streptomyces* genus are the most prolific source of novel compounds of medical and industrial relevance as antibiotic and anticancer drugs urgently needed to overcome clinical resistance and toxicity problems. Although traditionally considered soil bacteria, there is increasing evidence that *Streptomyces* species are ubiquitous, being present not only on terrestrial ecosystems, but also in some of the most extreme and less explored environments on our planet such as the oceans and the atmosphere.

New trends in drug discovery include the search for novel bioactive Actinobacteria in unexplored or underexplored environments. Previous reports in oceanic and atmospheric environments of the Cantabrian Sea region (North Spain, Northeast Atlantic) revealed that phylogenetically diverse Actinobacteria, with a great pharmacological potential, are widespread among intertidal and subtidal seaweeds (Braña et al., 2015; Sarmiento-Vizcaíno et al., 2016) and also among deep-sea coral reefs ecosystems (Sarmiento-Vizcaíno et al., 2017b), where a novel barotolerant actinobacterium, *Myceligenerans cantabricum*, was isolated (Sarmiento-Vizcaíno et al., 2015). Some of these marine strains were the source of nine new biologically active natural products with antibiotic properties against clinically relevant antibiotic resistant pathogens and cytotoxic activities toward diverse human cancer cell lines (Braña et al., 2017a, b; Sarmiento-Vizcaíno et al., 2017b; Ortiz-López et al., 2018; Rodríguez et al., 2018).

Strains belonging to three *Streptomyces* species widespread among these coastal and deep-sea habitats (*Streptomyces cyaneofuscatus, Streptomyces carnosus*, and *Streptomyces albidoflavus*) were also isolated from different cloud precipitation events happened in the Cantabrian Sea Coast (Braña et al., 2015; Sarmiento-Vizcaíno et al., 2016). Since then, atmospheric precipitations (hailstone, rainwater and snow) were used as natural sampling tools for the study of actinobacterial diversity in the atmosphere. Bioactive strains corresponding to about 3–4% of known *Streptomyces* species were isolated after precipitations and found to produce a great number of natural products with different biological activities, mainly as antimicrobial and anticancer agents (Sarmiento-Vizcaíno et al., 2018). These atmospheric-derived strains also produced 38 molecules not found in Natural products databases, thus revealing the atmosphere as a novel and promising source for natural product discovery.

Based on previous observations of cultivable *Streptomyces* species isolated in recent years from different precipitation events on the Cantabrian coast, an atmospheric dispersal model was proposed to explain the cosmopolitan distribution of highly halotolerant *Streptomyces* species (Sarmiento-Vizcaíno et al., 2016). According to this model, coupled to the Earth’s hydrological cycle, marine bioaerosols forming clouds contribute to the transfer of *Streptomyces* from oceans into the atmosphere, were they travel dispersed by winds, falling down to the earth by precipitation. Further support for this model came from a culture-independent approach, which reported Actinobacteria among the most dominant phyla in atmospheric precipitations in Japan, also showing seasonal variations in correlation with estimated air mass trajectories (Hiraoka et al., 2017). Connections between oceans, clouds and atmosphere, and their relevance in atmospheric chemistry and climate were addressed through the study of sea spray aerosols (Cochran et al., 2017). Actinobacterial transfer into sea spray aerosols in an experimental ocean-atmosphere mesocosm was also reported (Michaud et al., 2018).

In a culture dependent approach, we provide here further insights into the phylogenetic and secondary metabolic diversity of bioactive atmospheric Actinobacteria isolated from rainwater in precipitations events from Westerly winds in Spain over 4 years’ time. This approach involved rainwater sampling from different locations in Spain, meteorological analyses, taxonomical and phylogenetic analyses with identification at species level. Antimicrobial assays, metabolic profiling and LC-UV-MS analyses of compounds produced were used to assess the Actinobacteria biosynthetic diversity.

## Materials and Methods

### Sampling of Atmospheric Precipitations

**FIGURE 1:**
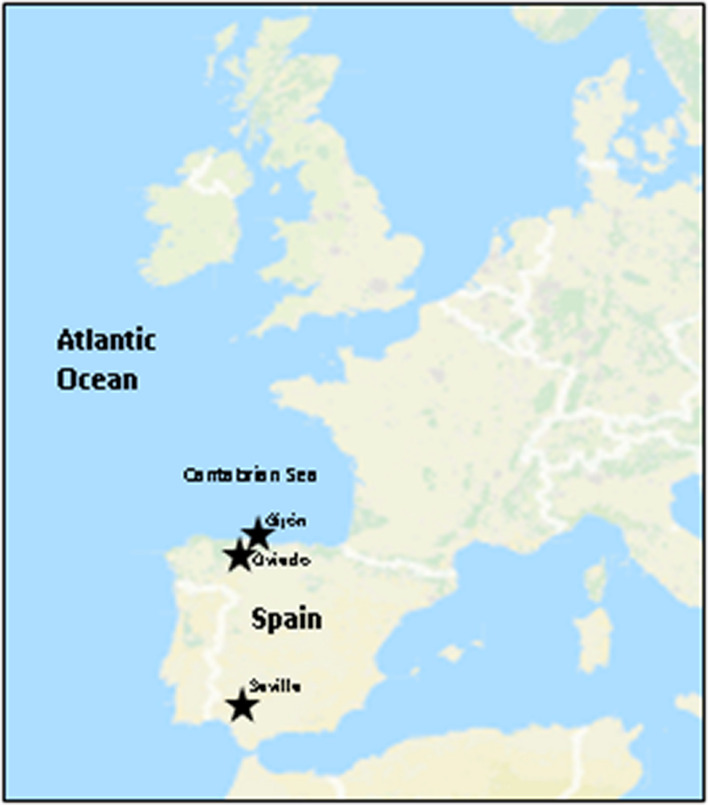
Sampling locations in Spain. Overview of the European Seas (Atlantic Ocean). Stars indicate the sampling locations in Northern and Southern Spain.

Atmospheric precipitations samples, including rainwater, hailstone and snow were collected within years 2013–2016 at the North of Spain, at the Cantabrian Sea coastal region of Asturias (Figure 1). This is a remarkably wet and rainy region, whose climate is under the influence of the Atlantic Ocean. Samples of 2–3 mL were taken in sterile recipients at the localities of Gijón (43° 32′ N, 5° 39′ W), and Oviedo (43° 21′ N, 5° 52′ W) and plated on selective agar media as previously described (Braña et al., 2015; Sarmiento-Vizcaíno et al., 2016). An additional rain sample (50 mL) was collected in 2016, in Seville (37° 23′ N, 5° 59′ W), Andalusia, South of Spain. Seville has a Mediterranean climate and is considered one of the warmest cities in continental Europe. During all precipitation events sampled here the prevailing wind direction has been Western.

### Isolation of Actinobacteria Strains and Culture Media

A collection of cultivable Actinobacteria strains were obtained after plating of precipitation samples on selective agar media, prepared with cycloheximide (80 μg mL^–1^) as antifungal and nalidixic acid (20 μg mL^–1^) as anti-Gram negative bacteria, using MOPS BLEB 1/6 (Oxoid) basal medium as previously reported (Sarmiento-Vizcaíno et al., 2016). Two different media either prepared with distilled water or with a supplement of 3.5% NaCl were used in selection plates. After 2–3 weeks of incubation at 28°C, colonies were selected based on different morphological features and pigment production on R5A agar plates. Isolated pure cultures were conserved in 20% glycerol at both −20° and −70°C. For halotolerance studies, MOPS BLEB 1/6 (Oxoid) was used as the basal medium, adding NaCl at 0, 3.5, 7.0, and 10.5% (w/v) final concentrations. R5A medium was used for secondary metabolite production as previously described (Sarmiento-Vizcaíno et al., 2018).

### Bioactive Strains Selection

**FIGURE 2:**
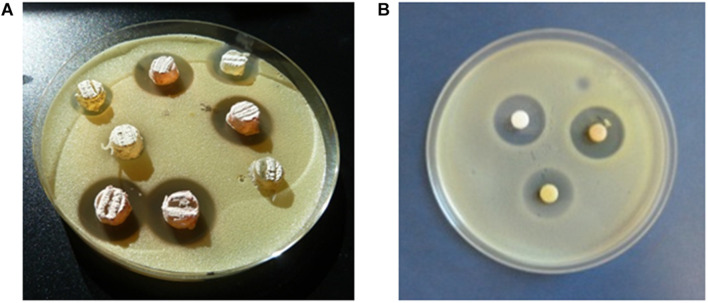
Bioassay diffusion assays. *Micrococcus luteus* was used as indicator microorganism. The zones of complete inhibition are measured as the diameters in mm. **(A)** Agar plugs. **(B)** AA discs loaded with ethyl acetate extracts of the isolates.

The antimicrobial activities of isolates were determined by agar diffusion methods using the following indicator microorganisms: the Gram-positive bacteria *Micrococcus luteus* ATCC 14452 and *Streptomyces* 85E ATCC 55824, the Gram-negative *Escherichia coli* ESS, and the yeast *Saccharomyces cerevisiae var. carlsbergensis* as previously reported (Sarmiento-Vizcaíno et al., 2018). Analyses were performed in TSA1/2 (Merck) against bacteria and in Sabouraud 1/2 (Pronadisa) against yeast. For antibiotic production Actinobacteria cultures were routinely cultured. Figure 2 shows an example of bioassays performed against *Micrococcus luteus* as indicator bacteria. Agar plugs of 7 mm diameter from Actinobacteria cultures on solid R5A medium (Figure 2A) were assay for initial selection of bioactive isolates. Also Kirby-Bauer based test using with 6-mm-diameter AA Discs (Whatman), loaded with ethyl acetate extracts of bioactive isolates, were performed (Figure 2B). Agar plugs assays detect all diffusible compounds produced by actinobacterial strains, both polar and apolar, whereas the AA discs bioassays only detect diffusible apolar molecules which were extracted with ethyl acetate.

### Air Mass Backward Trajectories Analyses

To estimate the long-range transport journey of air masses that originated the precipitation events herein studied, backward trajectories were generated using the HYSPLIT model (Hybrid Single Particle Lagrangian Integrated Trajectory) from the Global Data Assimilation System of National Oceanic and Atmospheric Administration, United States (Stein et al., 2015). To track the transport pathways of air masses and determine the origin of diverse air parcels, 5-day backward trajectories (used generally in bioaerosol studies) were obtained using the NOAA model.^1^ To find out the trajectories of atmospheric air masses, the sampling locations were used as the backward trajectory start point with altitudes over the sea level of 30, 1,000 and 3,000 m (Gijón), as previously reported (Sarmiento-Vizcaíno et al., 2018); 300, 1,000, and 3,000 m (Oviedo) and 7, 1,000, and 3,000 m (Seville).

### 16S RNA Analysis Identification and Phylogenetic Analysis

For taxonomic identification of the strains, DNA was extracted with a microbial isolation kit (Ultra Clean, MoBio Laboratories, Inc.) and standard methods were used for checking the purity (Russell and Sambrook, 2001). Partial 16S rRNA gene sequences of the bacterial strains were obtained by using the 616V (forward) and 699R (reverse) primers (Arahal et al., 2008) in PCR amplification as previously described (Braña et al., 2015). The nucleotide sequences were compared to sequences in databases using the BLAST program (Basic Local Alignment Search Tool) against the NCBI (National Centre for Biotechnology Information), submitted and deposited in the EMBL sequence database with accession numbers LR702033-LR702059. Phylogenetic analysis of the strains based on 16S rRNA sequences was performed as previously reported (Sarmiento-Vizcaíno et al., 2018).

### Chromatographic Analysis

Plugs of R5A plates (about 7 mL) were extracted using ethyl acetate in neutral and acidic (1% v/v formic acid) conditions. After evaporation, the organic fraction residue was redissolved in 100 μL of a mixture of DMSO and methanol (50:50). The analyses of the samples were performed by reversed phase liquid chromatography as previously described (Braña et al., 2015; Sarmiento-Vizcaíno et al., 2016).

### Identification of Compounds by LC-UV-Vis and LC-UV-HRMS Analyses

Samples were first analyzed and evaluated using an in-house HPLC-UV-Vis database. LC-UV-HRMS analyses were carried out as previously reported (Pérez-Victoria et al., 2016; Sarmiento-Vizcaíno et al., 2018) and major peaks in each chromatogram were searched against the MEDINA’s internal database and also against the Dictionary of Natural Products (DNP) (Chapman & Hall/CRC, 2015).

## Results

### Isolation and Characterization of Bioactive Atmospheric Actinobacteria by Sampling Multiple Precipitation Events in Spain

The strains herein studied were obtained from a unique Actinobacteria collection generated, during 4 years’ time frame (2013–2016) from diverse atmospheric precipitation events in Spain, as previously reported (Sarmiento-Vizcaíno et al., 2018). After a dereplication process involving phenotypical features, antibiotic activity and also meteorological analyses (see next section), 27 morphologically different bioactive strains isolated from rainwater from storm clouds transported by Western winds were selected for this study. Table 1 shows the results of initial antibiotic analyses of selected strains against a panel of indicator microorganisms (bacteria and fungi) by using agar diffusion assays (Figure 2A). The strains were isolated from samples collected in 12 rainfall events, and one hailstone event (A-241) at three different locations in Spain. The three different sampling places are shown in Figure 1. Among the 27 bioactive isolates, 18 were obtained from samples collected in the North Spain (43° N), 12 in the Cantabrian Sea coast (Gijón) and six strains at 28 km inland (Oviedo); finally 9 strains were isolated from a single rainfall event in South Spain (Seville, 37° N).

**TABLE 1:** Antibiotic activities of atmospheric Actinobacteria cultures against a panel of Gram-negative, Gram-positive bacteria and fungi.

Strain	*Escherichia coli*	*Micrococcus luteus*	*Streptomyces* 85E	*Saccharomyces cerevisiae*
A-43	−	12	11	−
A-50	−	22	10	−
A-53	−	13	18	−
A-69	−	−	10	−
A-87	11	24	11	16
A-139*	18	19	13	−
A−167	−	14	10	−
A-169	−	−	11	−
A-171	−	11	12	13
A-178	−	−	25	−
A-179	13	32	9	−
A-241	−	11	−	−
A-249	−	−	−	24
A-250	22	30	29	43
A-254	−	−	12	−
A-256	−	11	13	−
A-257	−	−	9	−
A-258	−	33	−	−
A-260	−	−	11	−
A-261	−	24	20	−
A-262	−	16	10	−
A-263	18	−	−	−
A-265	−	14	11	−
A-266	−	10	−	−
A-268	−	10	11	21
A-269	−	15	26	−
A-271	−	33	28	−

*The assays were initially performed with agar plugs from cultures and activities were estimated as the zones of complete inhibition (diameter in mm). The asterisk indicates that antibiotic activity was only detected in liquid cultures.*

### Backward Transport Trajectories Analyses

**FIGURE 3:**
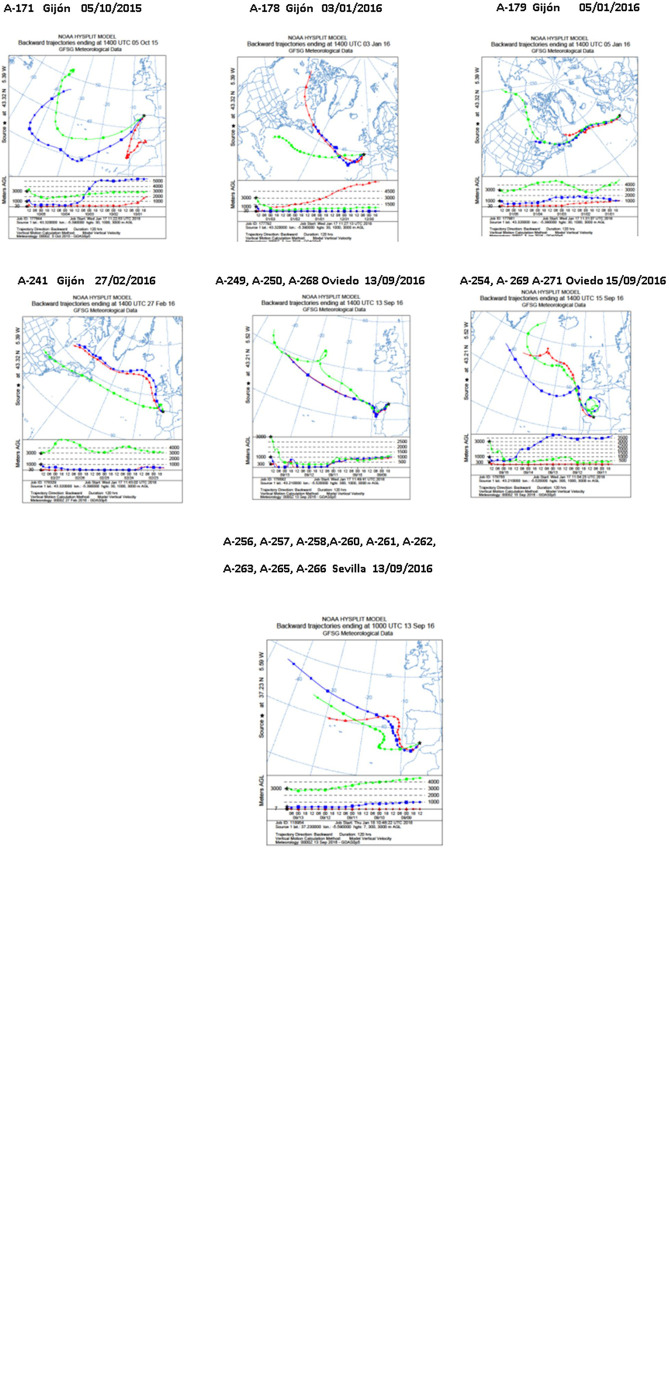
Five-day backward trajectories of air masses generating the storms that arrived at Spain and caused the diverse precipitation events. They were calculated with the NOAA HYSPLIT Model with three different transects with different arriving height as previously reported (Sarmiento-Vizcaíno et al., 2018). The sampling locations were used as the backward trajectory start point with altitudes over sea level of 30, 1,000, and 3,000 m (Gijón), 300, 1,000, and 3,000 m (Oviedo), and 7, 1,000, and 3,000 m (Seville). Sampling places are indicated by the black asterisks.

Meteorological analyses were performed to estimate the sources and trajectories of the different air masses that originated the precipitation events in which the selected strains were isolated. These sources were estimated using 5 days HYSPLIT backward trajectories. As shown in Figure 3, most backward trajectories showed air masses traveled eastward off the Atlantic Ocean toward continental Europe. As estimated, the air masses reaching the three sampling sites in Spain were predominantly of marine origin. In the atmospheric precipitation events herein studied, different air masses were transported by westerly winds (traveling at different altitudes) mainly from the Atlantic Ocean. In some events, that will be further stated, the trajectory also involves long-range transport from continental America, the Arctic Ocean and even the Northern Pacific Ocean, to downwind areas, such as the sampling place in continental Europe.

### Taxonomic Identification and Phylogenetic Analyses of Bioactive Isolates

**FIGURE 4:**
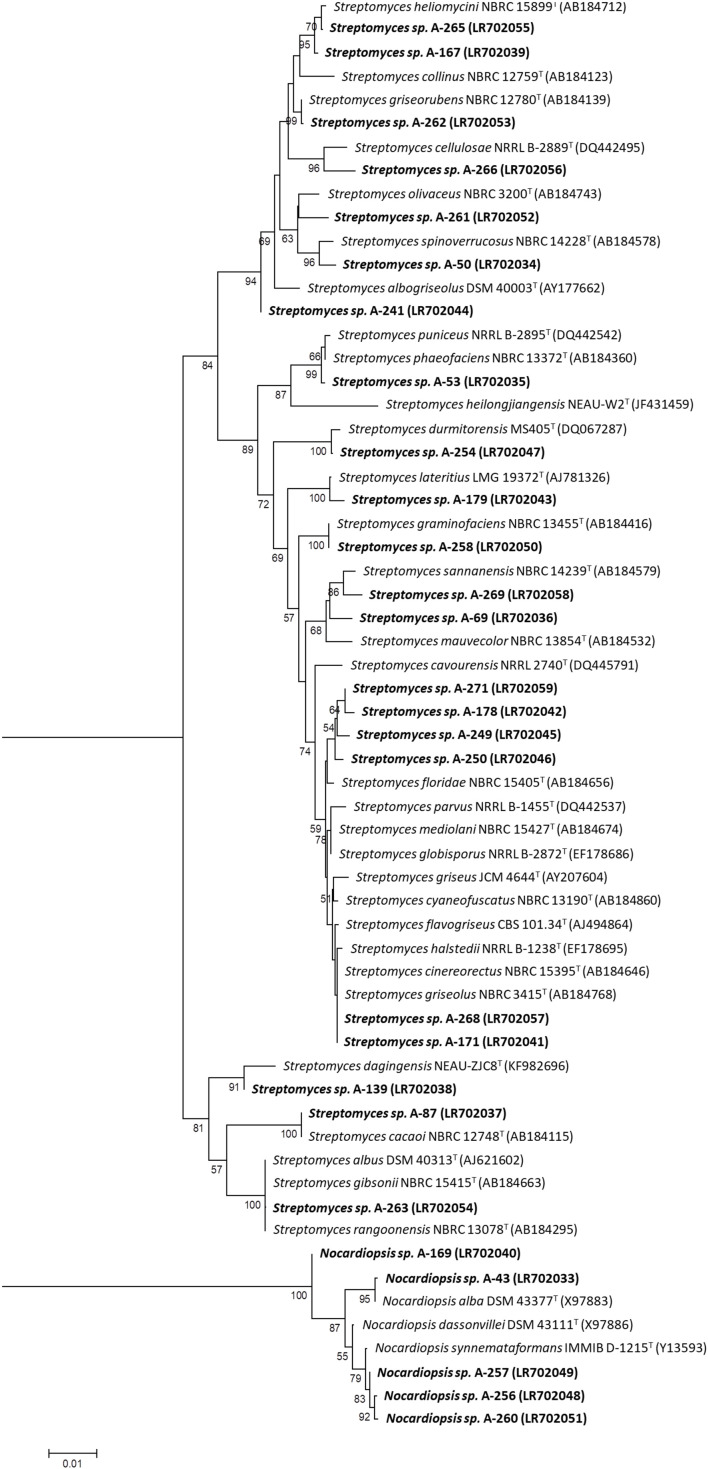
Neighbor-joining phylogenetic tree generated by distance matrix analysis of 16S rRNA gene sequences from atmospheric Actinobacteria (*Streptomyces* and *Nocardiopsis*) strains (highlighted) and nearest phylogenetic relatives. The numbers on branch nodes indicate bootstrap values (1,000 resamplings; only values > 50% are shown). Bar represents1% sequence divergence.

Identification of airborne-derived bioactive strains was determined by sequencing fragments of their 16S rRNA gene. Nucleotide sequences were then deposited in the EMBL database, and corresponding accession numbers are shown on Table 2. Phylogenetic analyses of isolates (Figure 4), based on 16S rRNA gene alignments, demonstrate that all isolates belong to two different genera among the *Phylum Actinobacteria*, since they share 99–100% identity with known actinobacterial species. As shown in Table 2, the identified strains have their closest homologs in previous species isolated from very diverse oceanic and terrestrial habitats. Among 27 studied isolates, 23 belonged to the *Streptomyces* genus, as previous reports in this environment. Interestingly, all these species are different from the ones isolated in a hailstone precipitation event from clouds transported by prevalent Northwestern winds (Sarmiento-Vizcaíno et al., 2018), thus suggesting that depending on the wind direction different strains can be isolated.

**TABLE 2:** Phylogenetic diversity of atmospheric-derived bioactive Actinobacteria isolates.

Strain	EMBL A. N.	NaCl%	Closest homolog	A. N.	% homology (bp)	Homolog isolation source	References
*Nocardiopsis* sp. A-43	LR702033	7	*Nocardiopsis alba* DSM 43377	X97883	100 (685/685)	Honeybees gut, United States; mushroom compost bioaerosol, Poland	Qiao et al., 2012; Paściak et al., 2014
*Streptomyces* sp. A-50	LR702034	3.5	*Streptomyces spinoverrucosus* NBRC 14228	AB184578	99.8 (985/987)	Marine	Hu et al., 2012;
*Streptomyces* sp. A-53	LR702035	3.5	*Streptomyces phaeofaciens* NBRC 13372	AB184360	99.7 (765/767)	Soil, Japan	Okamoto et al., 1986
*Streptomyces* sp. A-69	LR702036	3.5	*Streptomyces sannanensis* NBRC 14239	AB184579	99.7 (971/974)	Fresh water lake habitat, India	Singh et al., 2014
*Streptomyces* sp. A-87	LR702037	10.5	*Streptomyces cacaoi* NBRC 12748	AB184115	100 (993/993)	Cacao beans	Shirling and Gottlieb, 1968
*Streptomyces* sp. A-139	LR702038	3.5	*Streptomyces daqingensis* NEAU-ZJC8	KF982696	99.5 (764/768)	Saline-alkaline soil, China	Pan et al., 2016
*Streptomyces* sp. A-167	LR702039	7	*Streptomyces heliomycini* NBRC 15899	AB184712	99.8 (988/990)	Marine-derived, Saudi Arabia	Wang et al., 2017
*Nocardiopsis* sp. A-169	LR702040	7	*Nocardiopsis synnemataformans* IMMIB D-1215T	Y13593	99.3 (987/994)	Marine, terrestrial	Bennur et al., 2015
*Streptomyces* sp. A-171	LR702041	7	*Streptomyces griseolus* NBRC 3415	AB184768	100 (964/964)	Soil, Russia	Grammatikova et al., 2003
*Streptomyces*sp. A-178	LR702042	7	*Streptomyces cyaneofuscatus* 2–6	KJ571029	99.7 (959/962)	Marine, terrestrial and atmospheric, Spain	Sarmiento-Vizcaíno et al., 2016; 2018
*Streptomyces* sp. A-179	LR702043	3.5	*Streptomyces lateritius*LMG 19372	AJ781326	99.8 (969/971)	Soil	Elson et al., 1988
*Streptomyces* sp. A-241	LR702044	3.5	*Streptomyces collinus* NBRC 12759	AB184123	99.9 (710/711)	Soil, Germany	Rather et al., 2013
*Streptomyces* sp. A-249	LR702045	7	*Streptomyces griseolus* 11–11	KJ571072	99.9 (961/962)	Soil	Harder et al., 1991
*Streptomyces* sp. A-250	LR702046	3.5	*Streptomyces floridae* NBRC 15405	AB184656	99.8 (950/952)	Soil, Himalaya	Hussain et al., 2018
*Streptomyces* sp. A-254	LR702047	3.5	*Streptomyces durmitorensis*MS405	DQ067287	99.9 (974/975)	Soil, Serbia and Montenegro	Savic et al., 2007
*Nocardiopsis* sp. A-256	LR702048	10.5	*Nocardiopsis synnemataformans* IMMIB D-1215T	Y13593	99.3 (987/994)	Marine, terrestrial	Bennur et al., 2015
*Nocardiopsis* sp. A-257	LR702049	10.5	*Nocardiopsis synnemataformans* IMMIB D-1215T	Y13593	100 (1002/1002)	Marine, terrestrial	Bennur et al., 2015
*Streptomyces* sp. A-258	LR702050	3.5	*Streptomyces graminofaciens* NBRC 13455	AB184416	100 (968/968)	Soil, Japan	Fukuchi et al., 1995
*Nocardiopsis* sp. A-260	LR702051	7	*Nocardiopsis synnemataformans* IMMIB D-1215T	Y13593	100 (978/978)	Marine, terrestrial	Bennur et al., 2015
*Streptomyces* sp. A-261	LR702052	7	*Streptomyces albogriseolus* DSM 40003	AY177662	100 (977/977)	Sea sediment, China Sea	Cui et al., 2007
*Streptomyces* sp. A-262	LR702053	7	*Streptomyces griseorubens* NBRC 12780	AB184139	100 (965/965)	Soil, China	Xu and Yang, 2010
*Streptomyces* sp. A-263	LR702054	7	*Streptomyces albus* NRRL B-1811	NR118467	100 (990/990)	Atmosphere, soil, marine sediment, Spain	Sarmiento-Vizcaíno et al., 2018; Schleissner et al., 2011; Labeda et al., 2014
*Streptomyces* sp. A-265	LR702055	3.5	*Streptomyces heliomycini* 173574	EU593729	99.7 (978/981)	Marine-derived Saudi Arabia	Wang et al., 2017
*Streptomyces* sp. A-266	LR702056	7	*Streptomyces cellulosae* NRRL B-2889T	DQ442495	99.9 (991/992)	Soybean root	Liu et al., 2013
*Streptomyces* sp. A-268	LR702057	7	*Streptomyces griseolus* NBRC 3415	AB184768	99.9 (963/964)	Soil, Russia	Grammatikova et al., 2003
*Streptomyces* sp. A-269	LR702058	3.5	*Streptomyces sannanensis* NBRC 14239	AB184579	99.3 (949/956)	Fresh water lake habitat, India	Singh et al., 2014
*Streptomyces* sp. A-271	LR702059	3.5	*Streptomyces griseus* TBGT	KX269853	99.7 (950/953)	Soil; Mariana Trench sediment (10,898 m), Pacific Ocean	Goodfellow and Williams, 1983; Pathom-Aree et al., 2006

In addition, isolates belonging to the actinobacterial genus *Nocardiopsis* were herein identified in two precipitation events. A *Nocardiopsis alba* homolog, isolated in one of the North sampling places (Gijón), and several *Nocardiopsis synnemataformans* homologs in the South sampling place (Seville), which differ approximately in 6 latitudinal degrees. *Nocardiopsis* species were previously reported both in terrestrial and aquatic ecosystems (Bennur et al., 2015; Table 2) and are considered of pharmaceutical and biotechnological relevance due to its ability to produce diverse bioactive secondary metabolites (Bennur et al., 2016; Ibrahim et al., 2018).

A generalized feature of all Actinobacteria here studied is their ability to tolerate high NaCl concentrations, in the range 3.5–10.5% (Table 2). This high halotolerance is in agreement with previous reports within *Streptomyces* (Sarmiento-Vizcaíno et al., 2018) and in *Nocardiopsis* species, which are considered as the most abundant halophilic actinobacteria (Hamedi et al., 2013).

### Metabolite Profiling Analysis and Identification of Bioactive Secondary Metabolites Produced

**FIGURE 5:**
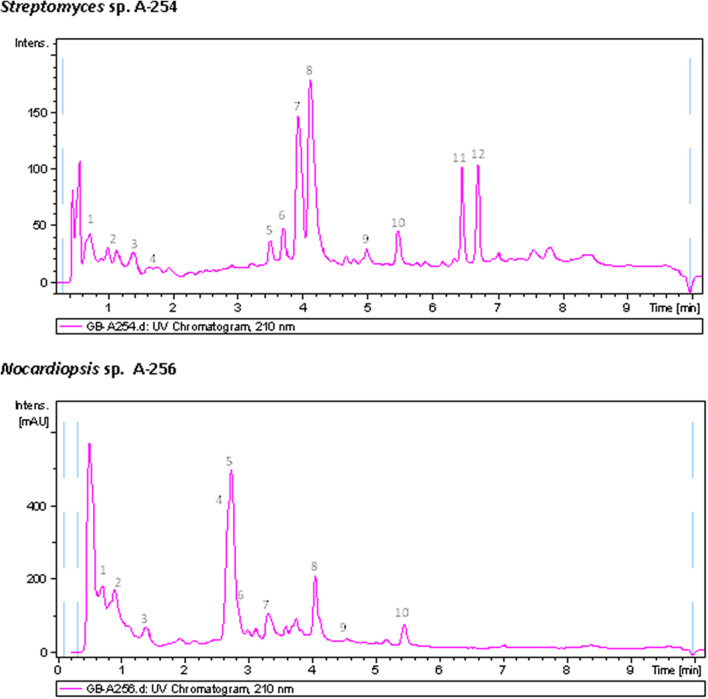
UV_210 nm_ chromatogram of samples A-254 and A-256 with peaks annotated showing dereplicated components. Dereplicated components in sample A-254: (1) Cyclo(prolylvalyl), (2) Cyclo(leucylprolyl), (3) Cyclo(phenylalanylprolyl), (4) Cyclo(prolytryptophyl), (5) C_21_H_38_NO_9_ (related to ravidomycin but with a molecular formula not found in the Dictionary of Natural Products), (6) Deacetylravidomycin, (7) C_30_H_33_NO_9_ (related to ravidomycin but with a molecular formula not found in the Dictionary of Natural Products), (8) Ravidomycin, (9) C_40_H_57_NO_10_ (molecular formula not found in the Dictionary of Natural Products), (10) Tetronomycin, (11) Salaceyin A and (12) Salaceyin B. Dereplicated components in sample A-256: (1) Bisucaberin B, (2) Cyclo(leucylprolyl), (3) Kahakamide A, (4) Endophenazine D, (5) Dihydroxyphenazine, (6) 1-Hydroxy-6-methoxyphenazine, (7) 1-Phenazinecarboxylic acid, (8) Piperafizine B, (9) 3-Benzylidene-6-(4-methoxybenzylidene)-2,5- piperazinedione, (10) 4′-Methoxyneihumicin or XR 330.

Chemical diversity of atmospheric Actinobacteria was assessed by metabolic profiling analyses of ethyl acetate extracts of bioactive strains, obtained in neutral and acidic conditions, screened for antibiotic production using agar diffusion with AA discs (Figure 2B), against a panel of indicator microorganisms (Table 3). Strong antibiotic activities were observed in all extracts, which were particularly active against *M. luteus*. The extracts were then analyzed for production of secondary metabolites by LC-UV and LC/HRMS analyses in combination with searches in UV and MS databases or the DNP after generation of a molecular formula of each peak based on HRMS results. Most of the strains show complex metabolic profiles producing multiple secondary metabolites in R5A medium (Supplementary Material 1). Figure 5 displays UV_210 *nm*_ chromatograms corresponding to *Nocardiopsis* sp. A-256 and *Streptomyces* sp. A-254 samples.

**TABLE 3:** Antibiotic activities of ethyl acetate extracts of the strains.

Strain	*Escherichia coli*	*Micrococcus luteus*	*Streptomyces* 85E	*Saccharomyces cerevisiae*
*Nocardiopsis sp.* A-43	−	17/19	ND	−
*Streptomyces* sp. A-50	−	20/19	ND	−
*Streptomyces* sp. A-53	−/8	14/22	ND	−
*Streptomyces* sp. A-69	−	9/−	ND	−
*Streptomyces* sp. A-87	10/9	24/24	ND	18/15
*Streptomyces* sp. A-139	−/18	−/19	ND	−
*Streptomyces* sp. A-167	13/−	11/−	−	−
*Nocardiopsis* sp. A-169	−	−	−	11/11
*Streptomyces* sp. A-171	18/−	13/−	25/26	−
*Streptomyces* sp. A-178	9/10	24/19	34/21	−
*Streptomyces* sp. A-179	−	10/−	−	−
*Streptomyces* sp. A-241	−	30/25	−	−
*Streptomyces* sp. A-249	ND	25/19	−/10	21/19
*Streptomyces* sp. A-250	ND	44/38	41/45	38/40
*Streptomyces* sp. A-254	ND	22/21	28/28	−
*Nocardiopsis* sp.A-256	ND	23/27	−	10/13
*Nocardiopsis* sp. A-257	ND	13/14	−	10/11
*Streptomyces* sp. A-258	ND	44/44	−	−
*Nocardiopsis* sp. A-260	−	−/12	−/13	−
*Streptomyces* sp. A-261	−	32/30	−/11	−
*Streptomyces* sp. A-262	ND	−/12	−	−
*Streptomyces* sp. A-263	17/18	−/12	−/10	−
*Streptomyces* sp. A-265	−	10/15	−	−/9
*Streptomyces* sp. A-266	−	19/17	−	−
*Streptomyces* sp. A-268	−	24/17	−	17/15
*Streptomyces* sp. A-269	−	30/31	−	−
*Streptomyces* sp. A-271	−/12	23/28	30/22	−

*Extracts obtained from 7 mL of culture, obtained in neutral and acidic conditions, were resuspended in 50 μL of DMSO-methanol (1:1) from which 15 μL were loaded onto AA discs. The discs were allowed to fully dry before applying to the indicator strain culture.*

Comparative analysis of *Streptomyces* and *Nocardiopsis* metabolites detected with natural product databases led to the identification of a total of 169 compounds detected after LC/MS dereplication in the ethyl acetate extracts of all strains metabolites, 139 were identified in the Dictionary of Natural Products, as shown in Table 4. Concerning the biological activity of identified natural products, the most frequent are antibiotics, with a total of 77 antibacterial and antifungal compounds, and also 32 antitumor or cytotoxic agents, 9 antiparasitic, 5 anti-inflammatory, 5 immunosuppressive, 3 antiviral, 2 insecticidal, 1 neuroprotective, 1 antiarthritic,1 plant hormone, 1 siderophore, 1 photoprotective and other products of diverse pharmacological and biotechnological relevance. Some compounds were only found to be produced by strains belonging to the *Nocardiopsis* genus, such as the antibacterial and anti*-Trypanosoma brucei* dihydroxyphenazine (A-256, A-257, A-260); the plant hormone Indol Acetic Acid (strain A-260), the antimicrobial kahakamide A, and the immunosuppressant N-(2-hydroxyphenyl)acetamide (A-257), among others.

**TABLE 4:** Identified compounds produced by atmospheric derived Actinobacteria strains and their biological activities.

Compound LC/MS	Strain	Biological activities
1-(2-Aminophenyl)ethanone/Phenylacetamide*	A-50	Antibacterial (Lu et al., 2020)
1-(Hydroxymethyl)-1H-indole-3-carboxylic acid	A-263	Antifouling (Wang et al., 2020)
1-Hydroxy-6-methoxyphenazine	**A-256, A-257**	Antimicrobial? Cook et al., 1971)
10-Oxide-1,8-Phenazinediol/5-Oxide-1,6-Phenazinediol/2,3,7-Phenazinetriol*	**A-260**	Antibiotic, antitumor, antimalaria, and antiparasitic activities (Laursen and Nielsen, 2004)
1-Methoxyphenazine	**A-257**	Antichlamydial activity (Bao et al., 2020)
1-Phenazinecarboxylic acid	**A-169, A-256**	Antifungal (Ye et al., 2010)
1-Phenazinol/2-Phenazinol*	**A-260**	Antibiotic (Vivian, 1956; Lu et al., 2013)
2,3,7-Phenazinetriol	**A-257**	Antibiotic, antitumor, antimalaria, and antiparasitic activities (Laursen and Nielsen, 2004)
2096D	A-263	Antiparasitic (Kelly et al., 2020)
2-(Acetoxymethyl)quinoline	A-179	Potential photoprotective (Sánchez-Suárez et al., 2020)
2-Hydroxy-1-(1H-indol-3-yl)ethanone/1H-Indole-3-carboxy Me ester/3-Indolylacetic acid/Skatole-2-carboxylic acid*	A-241	Antibacterial and antihelmintic (Himaja et al., 2010)
3-(Hydroxyacetyl)-1H-indole/1H-Indole-3-acetic acid/3-Methyl-1H-indole-2-carboxylic acid/Methyl 1H-Indole-3-carboxylate*	A-69, **A169**, A-258	Plant growth regulatory (Arteca, 1996)
35-Amino-32,33,34-bacteriohopanetriol	A-262	Sterol equivalent (Welander Paula et al., 2009)
3-Benzyl-6-isopropyl-2,5-piperazinedione	A-69	Unknown
3-Benzylidene-6-(3-hydroxy-2-methylpropylidene)-1-methyl-2,5-piperazinedione/Lansai C*	A-263	Anti-inflammatory (Thongchai et al., 2010)
3-Benzylidene-6-(4-methoxybenzylidene)-2,5-piperazinedione	**A-43, A-256**	
3-Indolylacetic acid	**A-260**	Plant hormone (Arteca, 1996)
3-Isobutylidene-6-(4-methoxybenzylidene)-2,5-piperazinedione	A-43	Antibiotic (Bycroft and Payne, 2013)
4,5-Dihydrogeldanamycin	A-120	Anticancer (Wu et al., 2012)
4-(5-Formyloxy-3-hydroxyhexyl)-3-methyl-2-oxetanone	A-266	Unknown
4-Hydroxy-2-methylquinazoline	**A-169**	Unknown
5-(6-Methyloctyl)-2(5H)-furanone/5-(6-Methyloctyl)-2(3H)-furanone/2,4,6-Trimethyl-2,4-decadienoic acid/5-Methyl-3-(5-methylheptyl)-2(5H)-furanone/11-Methyl-2,5-dodecadienoic acid*	A-265	Regulatory signal molecule (He et al., 2010)
5-Hydroxy-5-(hydroxymethyl)hexadecanoic acid	A-262	Unknown
6-(3-Methyl-2-butenyl)-1H-indole-3-acetaldehyde oxime	A-69	Unknown
8,10,12-Trihydroxy-2,4-dodecadienoic acid/4-(5-Formyloxy-3-hydroxyheptyl)-3-methyl-2-oxetanone/8,10,12-Trihydroxy-2,4-dodecadienoic acid*	A-271	Unknown
A 88696F/Jerangolide E/3,4-Dihydro-6,8-dihydroxy-3-tridecyl-1H-2-benzopyran-1-one*	A-262	Antifungal (Hans et al., 1997)
Actinonin	A-87	Anti-Gram-positive and Gram-negative foodborne pathogens (Jung et al., 2017)
Actiphenol	A-250	Antibiotic (Schrey et al., 2012)
Aggreceride A	A-262	Platelet aggregation inhibitor (Omura et al., 1986)
Aggreceride B	A-262	Platelet aggregation inhibitor (Omura et al., 1986)
Alaninolysine	**A-260**	Unknown
Albocycline	A-269	Antibiotic (Nagahama et al., 1967)
Albocycline M1/M2/M4/M5/M7*	A-269	Antibiotic (Managamuri et al., 2017)
Albocycline M3/M6*	A-269	Antibiotic (Bycroft and Payne, 2013)
Albonoursin	A-263	Antibiotic, antitumor (Fukushima et al., 1973)
Alkyldihydropyrone B/Alkyldihydropyrone A/Cyclohomononactic acid/1,3-Dihydroxy-4-methyl-6,8-decadien-5-one*	A-261	Cytotoxic against the leukemia cell lines (Aizawa et al., 2014); antifungal (Stadler et al., 2001)
Alteramide A	A-249, A-268	Cytotoxic (Shigemori et al., 1992); antifungal (Moree et al., 2014)
Alteramide B	A-268	Antifungal (Ding et al., 2016)
Angumycinone A/Boshracin D/Aranciamycin H/Antibiotic YT 127/Gaudimycin A/Hatomarubigin F/Ochracenomicin A*	A-249	Antibiotic (Igarashi et al., 1995; Kharel et al., 2012; Park et al., 2014); anticancer (Luzhetskyy et al., 2008)
Anhydrocycloheximide	A-250	Antifungal (Sullia and Griffin, 1977)
Antibiotic AKD 2A	A-262	Antibiotic, both antibacterial and antifungal (Akeda et al., 1995)
Antibiotic DC 81/Caerulomycin G*	A-262	Antibiotic (Kim, 2013); Cytotoxic (Fu et al., 2011)
Antibiotic FD 991	A-250	Antibiotic (Bycroft and Payne, 2013)
Antibiotic L 156588	A-258	Gastrin and brain cholecystokinin antagonists (Lam et al., 1991)
Antibiotic LL-BH872α/Geralcin E/5-Methyl-2-oxo-4-imidazolidinehexanoic acid*	A-171	Antibiotic (Bianchi et al., 2003)
Antibiotic TMC 1A/B *	A-241	Antibiotic, moderate cytotoxicity (Kohno et al., 1996)
Antibiotic TMC 1F	A-241	Antibiotic, moderate cytotoxicity (Kohno et al., 1996)
Antibiotic WS 7338A	A-87	Antibiotic, endotelin receptor antagonist (Miyata et al., 1992
Antibiotic WS 9326A	A-50	Tachykinin antagonist (Hashimoto et al., 1992)
Antibiotic WS 9326B	A-50	Tachykinin antagonist (Hashimoto et al., 1992)
Aranciamycin E/1-Butyl-3,6,8-trihydroxyanthraquinone-2-carboxylic acid/Fridamycin E/Gaudimycin B/C/β1-Rhodomycinone/Komodoquinone B/2-O-Demethyl-8-demethoxy-10-deoxysteffimycinone*	A-249	Antitumor (Luzhetskyy et al., 2008); antibiotic (Chen et al., 2011; Bycroft and Payne, 2013)
Aranciamycin H/Boshracin D/Angumycinone A/Hatomarubigin F/Gaudimycin A/Antibiotic YT 127/Ochracenomicin A*	A-268	Antitumor (Luzhetskyy et al., 2008); antibiotic (Igarashi et al., 1995; Kawasaki et al., 2010)
Aureusimine B	A-69	Antibiotic, against *Staphylococcus aureus* biofilms (Secor et al., 2012)
Bafilomycin A1	A-249	Vacuolar-type ATPase inhibitor, apoptosis (Tan et al., 2018)
Bafilomycin A1/C1*	A-268	Vacuolar-type ATPase inhibitor, apoptosis (Tan et al., 2018); antifungal (Frändberg et al., 2000)
Bafilomycin B1/E*	A-249, A-268	Antifungal (Frändberg et al., 2000)
Bafilomycin C1	A-249	Antifungal (Frändberg et al., 2000)
Bafilomycin D	A-268	Antibiotic, cytotoxic (Vu et al., 2018)
Benzylcarbamic acid/Streptokordin/2-Acetamidophenol/4-hydroxyphenylacetaldoxime*	**A-260**	Cytotoxic (Jeong et al., 2006); antifungal, anti-inflammatory, antitumor, anti-platelet, anti-arthritic (Guo et al., 2020)
Christolane C/9-Hydroxystreptazolin/13-Hydroxystreptazolin/Cytoxazone*	A-262	Antibiotic (Gómez et al., 2012); cytokine modulator (Kakeya et al., 1998)
Cyclo(isoleucylprolyl)	A-178, A-241	Unknown
Cyclo(leucylprolyl)	Several strains^*A*^	Antibiotic, cytotoxic (Santos et al., 2015)
Cyclo(phenylalanylprolyl)	A-178	Antibiotic (Santos et al., 2020)
Cyclo(prolyltryptophyl)	Several strains^*B*^	Broad spectrum antibacterial activity (Blunt and Munro, 2008)
Cyclo(prolyltyrosyl)	A-261	Cytotoxic (Blunt and Munro, 2008)
Cyclo(prolylvalyl)	Several strains^*C*^	Antifungal (Kumar et al., 2014)
Cyclo(valylprolyl)	A-139	Antibacterial (Alshaibani et al., 2017)
Cycloheximide	A-250	Antifungal (Siegel et al., 1966)
Deacetylravidomycin	A-254	Light dependent antitumor and antibiotic (Greenstein et al., 1986)
Dihydro-3-hydroxy-3-(1-hydroxy-2,4-hexadienyl)-4-(hydroxymethyl)-2(3H)-furanone/Xanthocidin*	A-262	Antibiotic (Asahi et al., 1966)
Dihydro-4-(hydroxymethyl)-3-(1-hydroxy-5-methylheptyl)-2(3H)-furanone/Dihydro-4-(hydroxymethyl)-3-(1-hydroxy-6-methylheptyl)-2(3H)-furanone/Dihydro-5-(hydroxymethyl)-3-(1-hydroxy-6-methylheptyl)-2(3H)-furanone/Dihydro-4-(hydroxymethyl)-3-(1-hydroxyoctyl)-2(3H)-furanone*	A-171	Antibiotic (Bycroft and Payne, 2013)
Dihydro-5-(6-hydroxy-6-methyloctyl)-2(3H)-furanone/7-Methoxy-4-dodecenoic acid*	A-262	Unknown
Dihydroxyphenazine	**A-256, A-257, A-260**	Antibacterial and anti-Trypanosoma brucei (Dashti et al., 2014)
Dinactin	A-266, A-271	Antibiotic (Silva et al., 2014); cytokine production inhibitor (Umland et al., 1999)
E 492	A-50	Anti-inflammatory (Ma et al., 2018)
E 975	A-50	Anti-inflammatory (Ma et al., 2018)
Echinomycin	A-250	Antitumor, antimicrobial (Kim et al., 2004)
Endophenazine D	A-256	Antibiotic (Gebhardt et al., 2002)
Feigrisolide C	A-266, A-271	Antiviral, antibacterial (Tang et al., 2000), antifungal against *Plasmopara viticola* zoospores (Islam et al., 2016)
Feigrisolide D	A-266, A-271	Antibacterial (Tang et al., 2000)
Ferrioxamine E	A-169	Siderophore (Berner et al., 1988)
Fumaramidmycin/N-[1-Hydroxy-2-(1H-indol-3-yl)-2-oxoethyl]acetamide*	A-50	Antibacterial (Maruyama et al., 1975)
Furanones	A-241	Antibiotic and antibiofilm (de Nys et al., 2006)
Geldanamycin	A-120	Antifungal, anticancer, neurotrophic and neuroprotective (Tadtong et al., 2007)
Germicidin A	A-53	Spore germination, hypha elongation (Aoki et al., 2011)
Germicidin D	A-50	Spore germination, hypha elongation (Aoki et al., 2011)
Glycerol 2-(15-methylhexadecanoate)/ Aggreceride C*	A-262	Platelet aggregation inhibitor (Omura et al., 1986)
Homononactic acid	A-271	Insecticidal (Jizba et al., 2008)
Ikarugamycin epoxide	A-249	Antibiotic against Gram-positive bacteria and fungi, strongly cytotoxic (Bertasso et al., 2003)
Ilamycin A/C1/C2	A-159	Cytotoxic (Ma et al., 2017)
Ilamycin B1	A-159, A-261	Unknown
JBIR 07/JBIR 08/N-Nonanoylhomoserine lactone/N-(7-Methyloctanoyl)homoserine lactone*	A-261	Autoinducer, signaling molecule (Patel et al., 2016)
Kahakamide A	**A-256**	Antimicrobial (Schumacher et al., 2001)
Lansai D	A-263	Anti-inflammatory (Taechowisan et al., 2010)
Lipoamide C	A-261	Antimicrobial (Berrue et al., 2009)
Lyngbic acid	A-268	Unknown
Maniwamycin A	A-171	Antifungal (Nakayama et al., 1989)
N-(2-hydroxyphenyl)acetamide	A-257	Immunosupressant (Jawed et al., 2010)
Pentaminomycin D	A-87	Autophagy inducer (Hwang et al., 2020)
Pentaminomycin E	A-87	Unknown
Methylsulfomycin I	A-105	Antibiotic (Vijaya Kumar et al., 1999)
Monactin	A-266, A-271	Antibiotic (Jizba et al., 1991)
N-Acetyl-4-hydroxybenzylamine/N-(2-Methoxyphenyl)acetamide/N-Methylphenylacetohydroxamic acid*	A-69	Unknown
N-Acetylisoleucine	A-265	Unknown
N-Acetyl-N-methyl-D-fucosamine	A-261	Unknown
N-Acetyltyramine	A-266	Antitumor human melanoma and leukemia (Kanou et al., 1998), Antifungal (Garcez et al., 2000), radical scavenging (Heidari and Mohammadipanah, 2018)
Narbosine B	A-171	Antiviral (Henkel et al., 1991)
Naseseazine A	A-87	Unknown
Naseseazine B	A-87	Antiplasmodial (Gomes et al., 2019
N-Butanoylhomoserine lactone	A-171, A-249	Quorum-sensing signal molecule in Gram-negative bacteria (Chan et al., 2011)
N-N-Dimethyladenosine	A-268	Inhibitor of AKT signaling in lung cancer cell lines (Vaden et al., 2017)
Non-actic acid	A-266, A-271	Antibiotic and antitumor (Meyers et al., 1965)
Non-actins	A-178, A-266, A-271	Ammonium ionophore, antibacterial, antiviral, antitumor (Zhan and Zheng, 2016)
O1,O2,O3,O4,N-Penta-Ac Valiolamine	A-139	Unknown
Ostreogrycin B	A-258	Antibiotic (Cocito, 1979)
Piperafizine B	A-169, A-256	Cytotoxicity potentiator (Kamei et al., 1990)
Prodigiosins	A-241	Antifungal, antimalarial, antitumor, immunosuppressive (Williamson et al., 2006; Stankovic et al., 2014; Darshan and Manonmani, 2015)
Questiomycin A/Crystalloiodinine B/1,8-Dihydroxyphenazine/1,9-Dihydroxyphenazine/2,3-Dihydroxyphenazine/1-Hydroxyphenazine 10-oxide*	A-169	Antibacterial (Shimizu et al., 2004), anticancer (Che et al., 2011)
Ravidomycin	A-254	Antibiotic, antitumor (Sehgal et al., 1983)
Respinomycin D	A-178	Antibiotic, antitumor (Ubukata et al., 1993)
Salaceyin A	A-254	Cytotoxic (Kim et al., 2006), antifungal (Park et al., 2007)
Salaceyin B	A-254	Cytotoxic (Kim et al., 2006), antifungal (Park et al., 2007)
Terferol (5′-Methoxy-[1,1′:4′,1′′-terphenyl]-2′,3′-diol)/3′-Methoxy-[1,1′:4′,1′′-terphenyl]-2′,6′-diol/3′-Methoxy-[1,1′:4′,1′′-terphenyl]-2′,5′-diol*	A-53	Unknown
Tetrahydro-5-methyl-6-(1-methylbutyl)-3-(2-methylpropyl)-2H-pyran-2-one/13-Methyl-4-tetradecenoic acid/12-Methyl-4-tetradecenoic acid*	A-258	Unknown
Tetranactin	A-266, A-271	Antibiotic, immunosuppressive and anti-proliferative (Tanouchi and Shichi, 1988)
Tetronomycin	A-254	Antibiotic (Keller-Juslén et al., 1982)
Tirandamycin A	A-171	Antiamoebic (Espinosa et al., 2012), antibiotic (Meyer, 1971)
Tirandamycin B	A-171	Antibiotic (Meyer, 1971)
Trinactin	A-266, A-271	Antibiotic, immunosuppressive (Tanouchi and Shichi, 1987)
Violapyrone F	A-241	Unknown
Virginiamycin M1	A-258	Antibiotic (Cocito, 1979)
Undecylprodigiosin	A-241	Antibiotic, cytotoxic (Petrović et al., 2017), immunosuppressor (Songia et al., 1997; Williamson et al., 2006)
Virginiamycin M2	A-258	Antibiotic (Cocito, 1979)
Xenocyloin C	A-261	Antibiotic (Paul et al., 1981), insecticidal (Proschak et al., 2014)
XR 330	A-43, A-256	Inhibitor of plasminogen activator inhibitor-1 activity (Bryans et al., 1996)
XR 334	A-169	Inhibitor of plasminogen activator inhibitor-1 activity (Bryans et al., 1996)
α,5-Dimethyl-2-oxo-4-imidazolidinehexanoic acid	A-171	Unknown
α-Methyldethiobiotin	A-50	Antibiotic (Hanka et al., 1972)

*The asterisk means that more than one compound was identified. The highlightened strains correspond to Nocardiopsis species, the rest are Streptomyces species. A: A-43, A-53, A-69, A-139, A-167, A-169, A-249, A-250, A-254, A-256, A-257, A-258, A-260, A-261, A-262, A-263, A-265, A-266, A-268, A-269, A-271. B: A-69, A-249, A-250, A-254, A-258, A268. C: A-241, A-250, A-254, A-258, A-269.*

Of great interest, 30 compounds had molecular formulae determined by HRMS not reported for any molecule included inNatural Products Databases(Supplementary Material 2). These molecules, 28 produced by *Streptomyces* species and two by *Nocardiopsis* sp. A-169, deserve further research since they might be new natural products and thus candidates for the discovery of new biologically active substances. Table 5 shows the number of identified compounds, the number of novel molecules produced by each strain, and the results of meteorological analyses to estimate the sources and trajectories of the different air masses that caused the precipitation events, estimated with a 5-day NOAA Hysplit Model (Figure 3). Concerning novel molecules, 20 were produced by strains isolated in the Northern Spain sampling places and 10 by strains isolated in Southern Spain. The air masses of the Southern precipitation event (strains A-258, A-261, A-262, A-266) originate in the Atlantic Ocean. The air masses corresponding to the Northern Spain precipitation events were also sourced in the Atlantic Ocean (strains A-167, A-169, A-249, and A-171), but in some cases (strains A-53, A-254, A-269, A-271) they originate in the Arctic Ocean, and continental America, strain A-87 in United States and strain A-139 in Canada.

**TABLE 5:** Number of compounds and sources of the producing Actinobacteria strains isolated from rainwater precipitations.

Strain	Number of products		Sampling place	Sampling date	Air masses backward trajectories analyses^*a*^
	**Unidentified**	**Identified**			
*Nocardiopsis* sp. A-43	5		Gijón	04/11/2013	California, United States South states from West to the East, Labrador (Canada), Atlantic Ocean.
*Streptomyces* sp. A-50		8	Gijón	19/12/2013	Northwest Passage (Artic Ocean), Atlantic Ocean, Spain
*Streptomyces* sp. A-53	2	3	Gijón	19/12/2013	Northwest Passage (Artic Ocean), Atlantic Ocean, Spain
*Streptomyces* sp. A-69		7	Gijón	15/12/2014	Pacific Ocean, Oregon, United States (from West to East), Terranova, Atlantic Ocean.
*Streptomyces* sp. A-87	1	6	Gijón	15/12/2014	Louisiana, Missisipi, Alabama, Georgia, South Carolina (United States), Atlantic Ocean, Labrador Terranova (Canada), Atlantic Ocean, Greenland, United Kingdom, France, Cantabrian Sea
*Streptomyces* sp. A-139	4	3	Gijón	18/01/2015	Manitoba, Ontario, Quebec, Terranova, Labrador (Canada), Atlantic Ocean, Arctic Ocean Iceland, Portugal, Spain
*Streptomyces* sp. A-167	2	3	Gijón	15/09/2015	Atlantic Ocean, Portugal, Spain
*Nocardiopsis* sp. A-169	2	9	Gijón	15/09/2015	Atlantic Ocean, Portugal, Spain
*Streptomyces* sp. A-171	1	8	Gijón	5/10/2015	Atlantic Ocean, Portugal, Mediterranean Sea
*Streptomyces* sp. A-178		4	Gijón	3/1/2016	Arctic Ocean (Baffin Bay), Hudson Bay, Quebec (Canada), Arctic Ocean, Atlantic Ocean, Portugal, Spain
*Streptomyces* sp. A-179		1	Gijón	5/1/2016	Pacific Ocean, Alaska (United States), North East Canada, Atlantic Ocean
*Streptomyces* sp. A-241		6	Gijón	27/02/2016	Michigan, New York, Maine (United States), Quebec (Canada), Atlantic Ocean
*Streptomyces* sp. A-249	1	11	Oviedo	13/09/2016	Atlantic Ocean, Portugal, Spain
*Streptomyces* sp. A-250		11	Oviedo	13/09/2016	Atlantic Ocean, Portugal, Spain
*Streptomyces* sp. A-254	3	9	Oviedo	15/09/2016	Arctic Ocean, Atlantic Ocean, Cantabrian Sea
*Nocardiopsis* sp. A-256		10	Seville	13/09/2016	Atlantic Ocean, Spain
*Nocardiopsis* sp. A-257		6	Seville	13/09/2016	Atlantic Ocean, Spain
*Streptomyces* sp. A-258	3	10	Seville	13/09/2016	Atlantic Ocean, Spain
*Nocardiopsis* sp. A-260		7	Seville	13/09/2016	Atlantic Ocean, Spain
*Streptomyces* sp. A-261	4	9	Seville	13/09/2016	Atlantic Ocean, Spain
*Streptomyces* sp. A-262	2	13	Seville	13/09/2016	Atlantic Ocean, Spain
*Streptomyces* sp.A-263		6	Seville	13/09/2016	Atlantic Ocean, Spain
*Streptomyces* sp. A-265		4	Seville	13/09/2016	Atlantic Ocean, Spain
*Streptomyces* sp. A-266	1	12	Seville	13/09/2016	Atlantic Ocean, Spain
*Streptomyces* sp. A-268		11	Oviedo	13/09/2016	Atlantic Ocean, Portugal, North Spain
*Streptomyces* sp. A-269	2	7	Oviedo	15/09/2016	Arctic Ocean, Atlantic Ocean, Cantabrian Sea
*Streptomyces* sp. A-271	2	11	Oviedo	15/09/2016	Arctic Ocean, Atlantic Ocean, Cantabrian Sea

*^a^Summary of the backward trajectories estimated with a 5-day NOAA Hyspli Model as shown in Figure 3.*

## Discussion

Exploration of the diversity of Actinobacteria producing biologically active natural products in the atmosphere was herein addressed by sampling multiple precipitation events with prevalent Westerly winds over 4 years in different sampling sites in Spain. Most of the isolates obtained from rainwater samples tolerate high salt concentrations and are homologs of known species isolated from very diverse terrestrial and marine ecosystems throughout the planet, in places as deep as the Mariana Trench sediments (10,898 m depth) in the Pacific Ocean, and as high as the Himalaya Mountains (8,849 m) (Table 2). Taxonomic identification and phylogenetic analyses of the atmospheric-derived Actinobacteria reported here, revealed *Streptomyces* as the most dominant genus, thus increasing the number of cultivable *Streptomyces* species able to survive and disperse via the atmosphere. Bioactive members of the rare actinobacterial genus *Nocardiopsis* were also isolated homologous to two species, *Nocardiopsis alba* and *Nocardiopsis synnemataformans*. The global number of *Nocardiopsis* species described so far on Earth is estimated in 50–53.^2^

The most relevant feature of the atmospheric Actinobacteria strains studied is that they are producers of multiple chemically diverse secondary metabolites, as analyzed by LC-UV-MS. Ten of the strains produced more than ten compounds each, up to a maximum of 15 (Table 5). From a total of 169 compounds detected after LC/MS dereplication, 82.25% were identified in the Dictionary of Natural Products, whereas, remarkably, the remaining 17.75%, not found in DPN, might be new molecules and deserve further research. After a literature search, 55% of the identified compounds were found to be biologically active as antibiotics (both against Gram-positive and Gram- negative bacteria and against fungi) and 23% have antitumor or cytotoxic activities; compounds with antiparasitic, anti-inflammatory, immunosuppressive, antiviral, insecticidal, neuroprotective, antiarthritic and other diverse biological activities were also detected in the extracts. The number of the compounds produced by these strains is estimated to be much higher than the one presented here, since only diffusible apolar molecules produced in a single culture conditions were analyzed, and possible diffusible polar or volatile molecules were not studied.

Meteorological analyses of the air masses involving 5 days HYSPLIT backward trajectories indicate a main oceanic source from the North Atlantic Ocean and also terrestrial sources from continental North America and Western Europe. In some events even long-range transport from the Pacific and the Arctic Oceans were also estimated. These bacteria remain viable after their atmospheric transport by winds across oceans and continents at planetary level. They could travel downwind and be dispersed via the atmosphere during long periods of time before they fall down to earth by precipitation. These findings provide further support for the *Streptomyces* atmospheric dispersal cycle (Sarmiento-Vizcaíno et al., 2016), which is herein extended to other members of the phylum Actinobacteria, such as *Nocardiopsis* genus.

The *Streptomyces* species herein identified are different from the ones previously isolated in a North-western wind precipitation event, sampled in North Spain and sourced in West Greenland and North Iceland and Canada (Sarmiento-Vizcaíno et al., 2018), thus indicating the relevance of winds in *Streptomyces* biogeographical distribution. Also, different *Nocardiopsis* species were isolated in different sampling places, which approximately differ in 6 latitudinal degrees, 37° N in South Spain to 43° N in North Spain sampling place. Latitude has been shown to delineate *Streptomyces* biogeography patterns in North America terrestrial environments (Choudoir et al., 2016).

Our findings make evident that across time, during different precipitation events, and space, by changing the latitude of the sampling place, we can have access to a striking diversity of Actinobacteria producing an extraordinary reservoir of bioactive natural products from remote and very distant origins, thus highlighting the relevance of the atmosphere as a here and now stablished source for the discovery of novel compounds of relevance in medicine and biotechnology.

## Conclusion

Results here obtained on Actinobacteria isolated in rainwater from storm clouds transported by Western winds in Spain highlights the relevance of the atmosphere as a main source of diverse *Streptomyces* and *Nocardiopsis* species, and increases our knowledge of the biogeography of these Actinobacteria genera on Earth. Our findings included also an amazing reservoir of bioactive molecules produced by these Actinobacteria, and take another step forward on the potential of atmospheric precipitations for the discovery of natural products active as antibiotic and antitumor agents, among others.

## Data Availability Statement

The datasets presented in this study can be found in online repositories. The names of the repository/repositories and accession number(s) can be found in the article/Supplementary Material.

## Author Contributions

AS-V and GB isolated the strains. AS-V performed the bioactivity assays, taxonomic identification, and phylogenetic analyses of the strains, and extraction of compounds produced, and analyzed the compounds by LC-UV. GB analyzed the air masses backward trajectories. JM and FR performed the metabolite profiling analysis and identified the compounds produced by LC-MS. LG and GB conceived and coordinated the project. GB wrote the manuscript which has been revised and approved by all authors.

## Conflict of Interest

The authors declare that the research was conducted in the absence of any commercial or financial relationships that could be construed as a potential conflict of interest.

## Publisher’s Note

All claims expressed in this article are solely those of the authors and do not necessarily represent those of their affiliated organizations, or those of the publisher, the editors and the reviewers. Any product that may be evaluated in this article, or claim that may be made by its manufacturer, is not guaranteed or endorsed by the publisher.

## Footnotes

1
http://ready.arl.noaa.gov/hypub-bin/trajtype.pl?runtype=archive

2
https://www.ncbi.nlm.nih.gov/Taxonomy/Browser/wwwtax.cgi

## Supplementary Material

Supplementary Material 1 UV_210 nm_ chromatograms corresponding to all samples.

Supplementary Material 2 Compounds whose molecular formulae was not found in the Dictionary of natural products.

## References

[B1] Aizawa T. Kim S. Y. Takahashi S. Koshita M. Tani M. Futamura Y. (2014). Alkyldihydropyrones, new polyketides synthesized by a type III polyketide synthase from Streptomyces reveromyceticus. *J. Antibiot.* 67 819–823. 10.1038/ja.2014.80 24984800

[B2] Akeda Y. Shibata K. Ping X. Tanaka T. Taniguchi M. (1995). AKD-2A, B, C and D, new antibiotics from Streptomyces sp. OCU-42815. Taxonomy, fermentation, isolation, structure elucidation and biological activity. *J. Antibiot.* 48 363–368. 10.7164/antibiotics.48.363 7797436

[B3] Alshaibani M. Zin N. M. Jalil J. Sidik N. Ahmad S. J. Kamal N. (2017). Isolation, Purification, and Characterization of Five Active Diketopiperazine Derivatives from Endophytic Streptomyces SUK 25 with Antimicrobial and Cytotoxic Activities. *J. Microbiol. Biotechnol.* 27 1249–1256. 10.4014/jmb.1608.08032 28535606

[B4] Aoki Y. Matsumoto D. Kawaide H. Natsume M. (2011). Physiological role of germicidins in spore germination and hyphal elongation in Streptomyces coelicolor A3(2). *J. Antibiot.* 64 607–611. 10.1038/ja.2011.59 21792209

[B5] Arahal D. R. Sánchez E. Macián M. C. Garay E. (2008). Value of recN sequences for species identification and as a phylogenetic marker within the family “Leuconostocaceae”. *Int. Microbiol.* 11 33–39. 18683630

[B6] Arteca R. (1996). *Plant Growth Substances: Principles and Applications.* New York, NY: Chapman&Hall.

[B7] Asahi K. Nagatsu J. Suzuki S. (1966). Xanthocidin, a new antibiotic. *J. Antibiot.* 19 195–199. 5953300

[B8] Bao X. Liu Z. Ni M. Xia C. Xu S. Yang S. (2020). Synthesis and Assessment of 3-Substituted Phenazines as Novel Antichlamydial Agents. *Med. Chem.* 16 413–421. 10.2174/1573406415666190708145639 31284867

[B9] Bennur T. Kumar A. R. Zinjarde S. Javdekar V. (2015). *Nocardiopsis* species: Incidence, ecological roles and adaptations. *Microbiol. Res.* 174 33–47. 10.1016/j.micres.2015.03.010 25946327

[B10] Bennur T. Ravi Kumar A. Zinjarde S. S. (2016). JavdekarV. *Nocardiopsis* species: a potential source of bioactive compounds. *J. Appl. Microbiol.* 120 1–16. 10.1111/jam.12950 26369300

[B11] Berner I. Konetschny-Rapp S. Jung G. Winkelmann G. (1988). Characterization of ferrioxamine E as the principal siderophore of Erwinia herbicola (*Enterobacter* agglomerans). *Biol. Met.* 1 51–56. 10.1007/BF01128017 2978958

[B12] Berrue F. Ibrahim A. Boland P. Kerr R. G. (2009). Newly isolated marine Bacillus pumilus (SP21): A source of novel lipoamides and other antimicrobial agents. *Pure Appl. Chem.* 81 1027–1031.

[B13] Bertasso M. Holzenkämpfer M. Zeeck A. Stackebrandt E. Beil W. Fiedler H.-P. (2003). Ripromycin and other polycyclic macrolactams from Streptomyces sp. Tü 6239: taxonomy, fermentation, isolation and biological properties. *J. Antibiot.* 56 364–371. 10.7164/antibiotics.56.364 12817810

[B14] Bianchi G. Dallavalle S. Merlini L. Nasini G. Quaroni S. (2003). A new azoxyalkene from a strain of an actinomadura-like fungus. *Planta Med.* 69 574–576. 10.1055/s-2003-40633 12865985

[B15] Blunt J. Munro M. H. G. (2008). *Dictionary of marine naturalproducts, with CD-ROM.* Boca Raton, FL: Chapman & Hall/CRC Press.

[B16] Braña A. F. Fiedler H. P. Nava H. González V. Sarmiento-Vizcaíno A. Molina A. (2015). Two *Streptomyces* species producing antibiotic, antitumor, and anti-inflammatory compounds are widespread among intertidal macroalgae and deep-sea coral reef invertebrates from the central Cantabrian Sea. *Microb. Ecol.* 69 512–524. 25319239 10.1007/s00248-014-0508-0

[B17] Braña A. F. Sarmiento-Vizcaíno A. Osset M. Pérez-Victoria I. Martín J. de Pedro N. (2017a). a New Natural Product with Cytotoxic Activity Produced by *Streptomyces* sp. M-207 Associated with the Deep-Sea Coral *Lophelia pertusa*. *Mar. Drugs* 15:144. 10.3390/md15050144 28534807 PMC5450550

[B18] Braña A. F. Sarmiento-Vizcaíno A. Pérez-Victoria I. Martín J. Otero L. Palacios-Gutiérrez J. J. (2019). Desertomycin G, a New Antibiotic with Activity against *Mycobacterium tuberculosis* and Human Breast Tumor Cell Lines Produced by *Streptomyces althioticus* MSM3, Isolated from the Cantabrian Sea Intertidal Macroalgae Ulva sp. *Mar. Drugs* 17:md17020114. 10.3390/md17020114 30759848 PMC6409695

[B19] Braña A. F. Sarmiento-Vizcaíno A. Pérez-Victoria I. Otero L. Fernández J. Palacios J. J. (2017b). Branimycins B and C, Antibiotics Produced by the Abyssal Actinobacterium Pseudonocardia carboxydivorans M-227. *J. Nat. Prod.* 80 569–573. 10.1021/acs.jnatprod.6b01107 28169531

[B20] Bryans J. Charlton P. Chicarelli-Robinson I. Collins M. Faint R. Latham C. (1996). Inhibition of plasminogen activator inhibitor-1 activity by two diketopiperazines, XR330 and XR334 produced by Streptomyces sp. *J. Antibiot.* 49 1014–1021. 10.7164/antibiotics.49.1014 8968395

[B21] Bycroft B. W. Payne D. J. (2013). *Dictionary of Antibiotics and Related Substances*, 2nd Edn. Boca Raton, FL: CRC Press.

[B22] Chan K. G. Puthucheary S. D. Chan X. Y. Yin W. F. Wong C. S. Too W. S. (2011). Quorum sensing in Aeromonas species isolated from patients in Malaysia. *Curr. Microbiol.* 62 167–172. 10.1007/s00284-010-9689-z 20544198

[B23] Chapman & Hall/CRC (2015). *Dictionary of Marine Natural Products (DMNP 2014).* Boca Raton, FL: Chapman & Hall/CRC.

[B24] Che X. F. Zheng C. L. Akiyama S. Tomoda A. (2011). 2-Aminophenoxazine-3-one and 2-amino-4,4α-dihydro-4α,7-dimethyl-3H-phenoxazine-3-one cause cellular apoptosis by reducing higher intracellular pH in cancer cells. *Proc. Jpn. Acad. Ser. B Phys. Biol. Sci.* 87 199–213. 10.2183/pjab.87.199 PMC3149379 21558757

[B25] Chen Q. Mulzer M. Shi P. Beuning P. J. Coates G. W. O’Doherty G. A. (2011). De novo asymmetric synthesis of fridamycin E. *Org. Lett.* 13 6592–6595. 10.1021/ol203041b 22107019 PMC3476477

[B26] Choudoir M. J. Doroghazi J. R. Buckley D. H. (2016). Latitude delineates patterns of biogeography in terrestrial *Streptomyces*. *Environ. Microbiol.* 2016:13420. 10.1111/1462-2920.13420 27322415

[B27] Cochran R. E. Ryder O. S. Grassian V. H. Prather K. A. (2017). Sea Spray Aerosol: The Chemical Link between the Oceans, Atmosphere, and Climate. *AccChem. Res.* 50 599–604. 10.1021/acs.accounts.6b00603 28945390

[B28] Cocito C. (1979). Antibiotics of the virginiamycin family, inhibitors which contain synergistic components. *Microbiol. Rev.* 43 145–192. 117294 10.1128/mr.43.2.145-192.1979 PMC281470

[B29] Cook F. D. Edwards O. E. Gillespie D. C. Peterson E. R. (1971). *1 Hydroxy 6 Methoxy Phenazines. United States Patent 3609153.* Alexandria, VA: United States Patent and Trademark Office.

[B30] Cui C. B. Liu H. B. Gu J. Y. Gu Q. Q. Cai B. Zhang D. Y. (2007). Echinosporins as new cell cycle inhibitors and apoptosis inducers from marine-derived Streptomyces albogriseolus. *Fitoterapia* 78 238–240. 17376609 10.1016/j.fitote.2006.11.017

[B31] Darshan N. Manonmani H. K. (2015). Prodigiosin and its potentialapplications. *J. Food Sci. Technol.* 52 5393–5407. 26344956 10.1007/s13197-015-1740-4 PMC4554646

[B32] Dashti Y. Grkovic T. Abdelmohsen U. R. Hentschel U. Quinn R. J. (2014). Production of induced secondary metabolites by a co-culture of sponge-associated actinomycetes, Actinokineospora sp. EG49 and Nocardiopsis sp. RV163. *Mar. Drugs* 12 3046–3059. 10.3390/md12053046 24857962 PMC4052330

[B33] de Nys R. Givskov M. Kumar N. Kjelleberg S. Steinberg P. D. (2006). Furanones. *Prog. Mol. Subcell Biol.* 42 55–86. 10.1007/3-540-30016-3_2 16805438

[B34] Ding Y. Li Y. Li Z. Zhang J. Lu C. Wang H. (2016). Alteramide B is a microtubule antagonist of inhibiting Candida albicans. *Biochim. Biophys. Acta* 1860 2097–2106. 10.1016/j.bbagen.2016.06.025 27373684 PMC4961524

[B35] Elson A. L. Box S. J. Gilpin M. L. (1988). New quinone antibiotics of the granaticin type, isolated from Streptomyces lateritius. I. Production, isolation and properties. *J. Antibiot.* 41 570–572. 10.7164/antibiotics.41.570 3372363

[B36] Espinosa A. Socha A. M. Ryke E. Rowley D. C. (2012). Antiamoebic properties of the actinomycete metabolites echinomycin A and tirandamycin A. *Parasitol. Res.* 111 2473–2477. 10.1007/s00436-012-3019-2 22763704 PMC3491104

[B37] Frändberg E. Petersson C. Lundgren L. N. Schnürer J. (2000). Streptomyces halstedii K122 produces the antifungal compounds bafilomycin B1 and C1. *Can. J. Microbiol.* 46 753–758. 10941524

[B38] Fu P. Wang S. Hong K. Li X. Liu P. Wang Y. (2011). Cytotoxic bipyridines from the marine-derived actinomycete Actinoalloteichus cyanogriseus WH1-2216-6. *J. Nat. Prod.* 74 1751–1756. 21770434 10.1021/np200258h

[B39] Fukuchi N. Furihata K. Nakayama J. Goudo T. Takayama S. Isogai A. (1995). Rotihibins, novel plant growth regulators from Streptomyces graminofaciens. *J. Antibiot.* 48 1004–1010. 10.7164/antibiotics.48.1004 7592044

[B40] Fukushima K. Yazawa K. Arai T. (1973). Biological activities of albonoursin. *J. Antibiot.* 26 175–176. 10.7164/antibiotics.26.175 4783201

[B41] Garcez W. S. Martins D. Garcez F. R. Marques M. R. Pereira A. A. Oliveira L. A. (2000). Effect of spores of saprophytic fungi on phytoalexin accumulation in seeds of frog-eye leaf spot and stem canker-resistant and -susceptible soybean (Glycine m ax L.) cultivars. *J. Agric Food Chem.* 48 3662–3665. 10956166 10.1021/jf991146o

[B42] Gebhardt K. Schimana J. Krastel P. Dettner K. Rheinheimer J. Zeeck A. (2002). Endophenazines A-D, new phenazine antibiotics from the arthropod associated endosymbiont Streptomyces anulatus. I. Taxonomy, fermentation, isolation and biological activities. *J. Antibiot.* 55 794–800. 10.7164/antibiotics.55.794 12458768

[B43] Gomes N. G. M. Pereira R. B. Andrade P. B. Valentão P. (2019). Double the Chemistry, Double the Fun: Structural Diversity and Biological Activity of Marine-Derived Diketopiperazine Dimers. *Mar. Drugs* 17:551. 10.3390/md17100551 PMC6835637 31569621

[B44] Gómez C. Olano C. Palomino-Schätzlein M. Pineda-Lucena A. Carbajo R. J. Braña A. F. (2012). Novel compounds produced by Streptomyces lydicus NRRL 2433 engineered mutants altered in the biosynthesis of streptolydigin. *J. Antibiot.* 65 341–348. 10.1038/ja.2012.37 22569159

[B45] Goodfellow M. Williams S. T. (1983). Ecology of Actinomycetes. *Annu. Rev. Microbiol.* 37 189–216. 10.1146/annurev.mi.37.100183.001201 6357051

[B46] Grammatikova N. E. Bibikova M. V. Spiridonova I. A. Kabanov A. E. Katlinskĭ A. V. (2003). Streptomyces griseolus # 182–a novel organism producing oligomycin antibiotics. Taxonomy, fermentation, and isolation. *Antibiot. Khimioter.* 48 11–15. 14558413

[B47] Greenstein M. Monji T. Yeung R. Maiese W. M. White R. J. (1986). Light-dependent activity of the antitumor antibiotics ravidomycin and desacetylravidomycin. *Antimicrob. Agents Chemother.* 29 861–866. 10.1128/aac.29.5.861 3729344 PMC284168

[B48] Guo S. Wang Y. Wang W. Hu H. Zhang X. (2020). Identification of new arylamine N-acetyltransferases and enhancing 2-acetamidophenol production in *Pseudomonas* chlororaphis HT66. *Microb. Cell Fact.* 19:105. 10.1186/s12934-020-01364-7 32430011 PMC7236291

[B49] Hamedi J. Mohammadipanah F. Ventosa A. (2013). Systematic and biotechnological aspects of halophilic and halotolerant actinomycetes. *Extremophiles* 17 1–13. 23129307 10.1007/s00792-012-0493-5

[B50] Hanka L. J. Martin D. G. Reineke L. M. (1972). Two new antimetabolites of biotin: alpha-methyldethiobiotin and alpha-methylbiotin. *Antimicrob. Agents Chemother.* 1 135–138. 10.1128/aac.1.2.135 4680803 PMC444181

[B51] Hans R. Höfle G. Gerth K. Washausen P. (1997). *Heterocyclic compounds obtainable from Sorangium cellulosum bacteria, their preparation process, and agents containing these compounds. Patent WO1997031912A1.* Geneva: WIPO.

[B52] Harder P. A. O’Keefe D. P. Romesser J. A. Leto K. J. Omer C. A. (1991). Isolation and characterization of Streptomyces griseolus deletion mutants affected in cytochrome P-450-mediated herbicide metabolism. *MGG Mol. General Genet.* 227:BF00259676. 10.1007/BF00259676 2062304

[B53] Hashimoto M. Hayashi K. Murai M. Fujii T. Nishikawa M. Kiyoto S. (1992). WS9326A, a novel tachykinin antagonist isolated from Streptomyces violaceusniger no. 9326. II. Biological and pharmacological properties of WS9326A and tetrahydro-WS9326A (FK224). 45 1064–1070. 10.7164/antibiotics.45.1064 1381344

[B54] He Y. W. Wu J. Cha J. S. Zhang L. H. (2010). Rice bacterial blight pathogen Xanthomonas oryzae pv. oryzae produces multiple DSF-family signals in regulation of virulence factor production. *BMC Microbiol.* 10:187. 10.1186/1471-2180-10-187 20615263 PMC2909994

[B55] Heidari B. Mohammadipanah F. (2018). Isolation and identification of two alkaloid structures with radical scavenging activity from Actinokineospora sp. UTMC 968, a new promising source of alkaloid compounds. *Mol. Biol. Rep.* 45 2325–2332. 10.1007/s11033-018-4395-1 30242664

[B56] Henkel T. Breiding-Mack S. Zeeck A. Grabley S. Hammann P. E. Hutter K. (1991). Liebigs. *Ann. Chem.* 1991:575.

[B57] Himaja M. Tesmine Jose M. V. Ramana R. A. Munirajasekhar D. (2010). Synthesis and biological evaluation of indole-3-carboxylic acid derivatives of amino acids and peptides. *Int. Res. J. Pharmacy* 1 436–440.

[B58] Hiraoka S. Miyahara M. Fujii K. Machiyama A. Iwasaki W. (2017). Seasonal Analysis of Microbial Communities in Precipitation in the Greater Tokyo Area, Japan. *Front. Microbiol.* 8:1506. 10.3389/fmicb.2017.01506 28848519 PMC5554504

[B59] Hu Y. Martinez E. D. MacMillan J. B. (2012). Anthraquinones from a marine-derived Streptomyces spinoverrucosus. *J. Nat. Prod.* 75 1759–1764. 10.1021/np3004326 23057874 PMC3488424

[B60] Hussain A. Rather M. A. Dar M. S. Dangroo N. A. Aga M. A. Qayum A. (2018). Streptomyces puniceus strain AS13., Production, characterization and evaluation of bioactive metabolites: A new face of dinactin as an antitumor antibiotic. *Microbiol. Res.* 207 196–202. 10.1016/j.micres.2017.12.004 29458855

[B61] Hwang S. Le L. T. H. L. Jo S. I. Shin J. Lee M. J. Oh D. C. (2020). Pentaminomycins C-E: Cyclic Pentapeptides as Autophagy Inducers from a Mealworm Beetle Gut Bacterium. *Microorganisms* 8:1390. 10.3390/microorganisms8091390 32927831 PMC7565604

[B62] Ibrahim A. H. Desoukey S. Y. Fouad M. A. Kamel M. S. Gulder T. A. M. Abdelmohsen U. R. (2018). Natural Product Potential of the Genus Nocardiopsis. *Mar. Drugs* 16:E147. 10.3390/md16050147 29710816 PMC5983278

[B63] Igarashi M. Sasao C. Yoshida A. Naganawa H. Hamada M. Takeuchi T. (1995). Ochracenomicins A, B and C, new benz[a]anthraquinone antibiotics from Amicolatopsis sp. *J. Antibiot.* 48 335–337. 10.7164/antibiotics.48.335 7775274

[B64] Islam M. T. Laatsch H. von Tiedemann A. (2016). Inhibitory Effects of Macrotetrolides from *Streptomyces* spp. On Zoosporogenesis and Motility of Peronosporomycete Zoospores Are Likely Linked with Enhanced ATPase Activity in Mitochondria. *Front. Microbiol.* 7:1824. 10.3389/fmicb.2016.01824 27917156 PMC5114239

[B65] Jawed H. Shah S. U. Jamall S. Simjee S. U. (2010). N-(2-hydroxy phenyl) acetamide inhibits inflammation-related cytokines and ROS in adjuvant-induced arthritic (AIA) rats. *Int. Immunopharmacol.* 10, 900–905. 10.1016/j.intimp.2010.04.028 20452462

[B66] Jeong S. Y. Shin H. J. Kim T. S. Lee H. S. Park S. K. Kim H. M. (2006). Streptokordin, a new cytotoxic compound of the methylpyridine class from a marine-derived Streptomyces sp. KORDI-3238. *J. Antibiot.* 59 234–240. 10.1038/ja.2006.33 16830891

[B67] Jizba J. Přikrylová V. Ujhelyiova L. Varkonda Š (2008). “Insecticidal properties of nonactic acid and homononactic acid, the precursors of macrotetrolide antibiotics.”. *Folia Microbiol.* 37 299–303.

[B68] Jizba J. Sedmera P. Zima J. Beran M. Blumauerová M. Kandybin N. V. (1991). Macrotetrolide antibiotics produced by Streptomyces globisporus. *Folia Microbiol.* 36 437–443. 10.1007/BF02884062 1821868

[B69] Jung D. Yum S. J. Jeong H. G. (2017). Characterization and evaluation of antimicrobial activity of actinonin against foodborne pathogens. *Food Sci. Biotechnol.* 26 1649–1657. 30263702 10.1007/s10068-017-0190-3 PMC6049706

[B70] Kakeya H. Morishita M. Kobinata K. Osono M. Ishizuka M. Osada H. (1998). Isolation and biological activity of a novel cytokine modulator, cytoxazone. *J. Antibiot.* 51 1126–1128. 10.7164/antibiotics.51.1126 10048575

[B71] Kamei H. Oka M. Hamagishi Y. Tomita K. Konishi M. Oki T. (1990). Piperafizines A and B, potentiators of cytotoxicity of vincristine. *J. Antibiot.* 43 1018–1020. 10.7164/antibiotics.43.1018 2211350

[B72] Kanou M. Ohta Y. Tanaka S. Yokoyama Y. Tanba M. Seawa T. (1998). *New compound, its production and anticancer agent, Japanese Kokai Tokkyo Koho (Japanese Patent), JP. 10259174.* Tokyo: Japan Patent Office.

[B73] Kawasaki T. Yamada Y. Maruta T. Maeda A. Hayakawa Y. (2010). Hatomarubigin E, a biosynthetic intermediate of hatomarubigins C and a substrate of HrbU O-methyltransferase. *J. Antibiot.* 63 725–727. 10.1038/ja.2010.118 20959847

[B74] Keller-Juslén C. King H. D. Kuhn M. Loosli H. R. Pache W. Petcher T. J. (1982). Tetronomycin, a novel polyether of unusual structure. *J. Antibiot.* 35 142–150. 10.7164/antibiotics.35.142 7076564

[B75] Kelly P. Hadi-Nezhad F. Liu D. Y. Lawrence T. J. Linington R. G. Ibba M. (2020). Targeting tRNA-synthetase interactions towards novel therapeutic discovery against eukaryotic pathogens. *PLoS Negl. Trop. Dis.* 14:e0007983. 10.1371/journal.pntd.0007983 32106219 PMC7046186

[B76] Kharel M. K. Pahari P. Shepherd M. D. Tibrewal N. Nybo S. E. Shaaban K. A. (2012). Angucyclines: Biosynthesis, mode-of-action, new natural products, and synthesis. *Nat. Prod. Rep.* 29 264–325. 10.1039/c1np00068c 22186970 PMC11412254

[B77] Kim N. Shin J. C. Kim W. Hwang B. Y. Hong Y. S. Lee D. (2006). Cytotoxic 6-alkylsalicylic acidsfrom the endophytic Streptomyces laceyi. *J. Antibiot.* 59 797–800. 10.1038/ja.2006.105 17323647

[B78] Kim S.-K. (ed.) (2013). *Marine microbiology: bioactive compounds and biotechnological applications.* Hoboken, NJ: Wiley.

[B79] Kim Y. B. Kim Y. H. Park J. Y. Kim S. K. (2004). Synthesis and biological activity of new quinoxaline antibiotics of echinomycin analogues. *Bioorg. Med. Chem. Lett.* 14 541–544. 10.1016/j.bmcl.2003.09.086 14698199

[B80] Kohno J. Nishio M. Kawano K. Nakanishi N. Suzuki S. Uchida T. (1996). TMC-1 A, B, C and D, new antibiotics of the manumycin group produced by Streptomyces sp. Taxonomy, production, isolation, physico-chemical properties, structure elucidation and biological properties. *J. Antibiot.* 49 1212–1220. 10.7164/antibiotics.49.1212 9031666

[B81] Kumar S. N. Nambisan B. Sundaresan A. Mohandas C. Anto R. J. (2014). Isolation and identification of antimicrobial secondary metabolites from Bacillus cereus associated with a rhabditid entomopathogenic nematode. *Ann. Microbiol.* 64 209–218. 10.1007/s13213-013-0653-6

[B82] Labeda D. P. Doroghazi J. R. Ju K. S. Metcalf W. W. (2014). Taxonomic evaluation of Streptomyces albus and related species using multilocus sequence analysis and proposals to emend the description of Streptomyces albus and describe Streptomyces pathocidini sp. nov. *Int. J. Syst. Evol. Microbiol.* 64(Pt 3), 894–900. 10.1099/ijs.0.058107-0 24277863 PMC4851252

[B83] Lam Y. K. Bogen D. Chang R. S. Faust K. A. Hensens O. D. Zink D. L. (1991). Novel and potent gastrin and brain cholecystokinin antagonists from Streptomyces olivaceus. Taxonomy, fermentation, isolation, chemical conversions, and physico-chemical and biochemical properties. *J. Antibiot.* 44 613–625. 10.7164/antibiotics.44.613 1906451

[B84] Laursen J. B. Nielsen J. (2004). Phenazine natural products: biosynthesis, synthetic analogues, and biological activity. *Chem. Rev.* 104 1663–1686. 10.1021/cr020473j 15008629

[B85] Liu C. Wang X. Yan Y. Wang J. Zhang B. Zhang J. (2013). Streptomyces heilongjiangensis sp. nov., a novel actinomycete that produces borrelidin isolated from the root surface of soybean [Glycine max (L.) Merr]. *Int. J. Syst. Evol. Microbiol.* 63(Pt 3), 1030–1036. 10.1099/ijs.0.041483-0 22707527 PMC3709533

[B86] Lu C. H. Li Y. Y. Wang H. X. Wang B. M. Shen Y. M. (2013). A new phenoxazine derivative isolated from marine sediment actinomycetes, Nocardiopsis sp. 236. *Drug Discov. Ther.* 7 101–104. 23917857

[B87] Lu H. Zhou X. Wang L. Jin L. (2020). Synthesis and Antibacterial Evaluation of N-phenylacetamide Derivatives Containing 4-arylthiazole Moieties. *Molecules* 25:1772. 10.3390/molecules25081772 32290634 PMC7221908

[B88] Luzhetskyy A. Hoffmann J. Pelzer S. Wohlert S. E. Vente A. Bechthold A. (2008). Aranciamycin analogs generated by combinatorial biosynthesis show improved antitumor activity. *Appl. Microbiol. Biotechnol.* 80 15–19. 10.1007/s00253-008-1515-1 18553079

[B89] Ma J. Cao B. Liu C. Guan P. Mu Y. Jiang Y. (2018). Actinofuranones D-I from a Lichen-Associated Actinomycetes, Streptomyces gramineus, and Their Anti-Inflammatory Effects. *Molecules* 23:2393. 10.3390/molecules23092393 30231581 PMC6225470

[B90] Ma J. Huang H. Xie Y. Liu Z. Zhao J. Zhang C. (2017). Biosynthesis of ilamycins featuring unusual building blocks and engineered production of enhanced anti-tuberculosis agents. *Nat. Commun.* 8:391. 10.1038/s41467-017-00419-5 28855504 PMC5577134

[B91] Managamuri U. Vijayalakshmi M. Ganduri V. S. R. K. Rajulapati S. B. Bonigala B. Kalyani B. S. (2017). Isolation, identification, optimization, and metabolite profiling of Streptomyces sparsus VSM-30. *3 Biotech.* 7:217. 10.1007/s13205-017-0835-1 28669076 PMC5494031

[B92] Maruyama H. B. Suhara Y. Suzuki-Watanabe J. Maeshima Y. Shimizu N. A. (1975). new antibiotic, fumaramidmycin I. Production, biological properties and characterization of producer strain. *J. Antibiot.* 28 636–647. 10.7164/antibiotics.28.636 1184477

[B93] Meyer C. E. (1971). Tirandamycin, a new antibiotic isolation and characterization. *J. Antibiot.* 24 558–560. 10.7164/antibiotics.24.558 5092790

[B94] Meyers E. Pansy F. E. Perlman D. Smith D. A. Weisenborn F. L. (1965). The in vitro activity of Nonactin and its homologs: Monactin, Dinactin and Trinactin. *J. Antibiot.* 18 128–129. 14336167

[B95] Michaud J. M. Thompson L. R. Kaul D. Espinoza J. L. Richter R. A. Xu Z. Z. (2018). Taxon-specific aerosolization of bacteria and viruses in an experimental ocean-atmosphere mesocosm. *Nat. Commun.* 9:2017. 10.1038/s41467-018-04409 PMC5964107 29789621

[B96] Miyata S. Hashimoto M. Masui Y. Ezaki M. Takase S. Nishikawa M. (1992). WS-7338, new endothelin receptor antagonists isolated from Streptomyces sp. No. 7338. I. Taxonomy, fermentation, isolation, physico-chemical properties and biological activities. *J. Antibiot.* 45 74–82. 10.7164/antibiotics.45.74 1312521

[B97] Moree W. J. McConnell O. J. Nguyen D. D. Sanchez L. M. Yang Y. L. Zhao X. (2014). Microbiota of healthy corals are active against fungi in a light-dependent manner. *ACS Chem. Biol.* 9 2300–2308. 10.1021/cb500432j 25058318 PMC4201335

[B98] Nagahama N. Suzuki M. Awataguchi S. Okuda T. (1967). Studies on a new antibiotic, albocycline. I. Isolation, purification and properties. *J. Antibiot.* 20 261–266. 5630768

[B99] Nakayama M. Takahashi Y. Itoh H. Kamiya K. Shiratsuchi M. Otani G. (1989). Novel antifungal antibiotics maniwamycins A and B. I. Taxonomy of the producing organism, fermentation, isolation, physico-chemical properties and biological properties. *J. Antibiot.* 42 1535–1540. 10.7164/antibiotics.42.1535 2584134

[B100] Okamoto M. Yoshida K. Nishikawa M. Ando T. Iwami M. Kohsaka M. (1986). FR-900452, a specific antagonist of platelet activating factor (PAF) produced by Streptomyces phaeofaciens. I. Taxonomy, fermentation, isolation, and physico-chemical and biological characteristics. *J. Antibiot.* 39 198–204. 10.7164/antibiotics.39.198 3082838

[B101] Omura S. Nakagawa A. Fukamachi N. Otoguro K. Kobayashi B. (1986). Aggreceride, a new platelet aggregation inhibitor from Streptomyces. *J. Antibiot.* 39 1180–1181. 10.7164/antibiotics.39.1180 3759668

[B102] Ortiz-López F. J. Alcalde E. Sarmiento-Vizcaíno A. Díaz C. Cautain B. García L. A. (2018). New 3-Hydroxyquinaldic Acid Derivatives from Cultures of the Marine Derived Actinomycete *Streptomyces cyaneofuscatus* M-157. *Mar. Drugs* 16:371. 10.3390/md16100371 30297652 PMC6212950

[B103] Pan T. He H. Li C. Zhao J. Zhang Y. Li J. (2016). Streptomyces daqingensis sp. nov., isolated from saline-alkaline soil. *Int. J. Syst. Evol. Microbiol.* 66 1358–1363. 10.1099/ijsem.0.000887 26755354

[B104] Park C. N. Lee D. Kim W. Hong Y. Ahn J. S. Kim B. S. (2007). Antifungal activity of salaceyin A against Colletotrichum orbiculare and Phytophthora capsici. *J. Basic Microbiol.* 47 332–339. 17647212 10.1002/jobm.200710325

[B105] Park H. B. Lee J. K. Lee K. R. Kwon H. C. (2014). Angumycinones A and B, two new angucyclic quinones from Streptomyces sp. KMC004 isolated from acidic mine drainage. *Tetrahed. Lett.* 55 63–66.

[B106] Paściak M. Pawlik K. Gamian A. Szponar B. Skóra J. Gutarowska B. (2014). An airborne actinobacteriaNocardiopsisalba isolated from bioaerosol of a mushroom compost facility. *Aerobiologia* 30 413–422. 25382928 10.1007/s10453-014-9336-4 PMC4218971

[B107] Patel N. M. Moore J. D. Blackwell H. E. Amador-Noguez D. (2016). Identification of Unanticipated and Novel N-Acyl L-Homoserine Lactones (AHLs) Using a Sensitive Non-Targeted LC-MS/MS Method. *PLoS One* 11:e0163469. 10.1371/journal.pone.0163469 27706219 PMC5051804

[B108] Pathom-Aree W. Stach J. E. Ward A. C. Horikoshi K. Bull A. T. Goodfellow M. (2006). Diversity of actinomycetes isolated from Challenger Deep sediment (10,898 m) from the Mariana Trench. *Extremophiles* 10 181–189. 10.1007/s00792-005-0482-z 16538400

[B109] Paul V. J. Frautschy S. Fenical W. Nealson K. H. (1981). Antibiotics in microbial ecology : Isolation and structure assignment of several new antibacterial compounds from the insect-symbiotic bacteriaXenorhabdus spp. *J. Chem. Ecol.* 7 589–597. 10.1007/BF00987707 24420598

[B110] Pérez-Victoria I. Martín J. Reyes F. (2016). Combined LC/UV/MS and NMR Strategies for the Dereplication of Marine Natural Products. *Planta Med.* 82 857–871. 27002401 10.1055/s-0042-101763

[B111] Petrović S. Vasić V. Mitrović T. Lazović S. Leskovac A. (2017). The impact of concentration and administration time on the radiomodulating properties of undecylprodigiosin in vitro. *Arh. Hig. Rada Toksikol.* 68 1–8. 10.1515/aiht-2017-68-289 28365670

[B112] Proschak A. Zhou Q. Schöner T. Thanwisai A. Kresovic D. Dowling A. (2014). Biosynthesis of the insecticidal xenocyloins in Xenorhabdus bovienii. *Chembiochem* 15 369–372. 10.1002/cbic.201300694 24488732

[B113] Qiao J. Chen L. Li Y. Wang J. Zhang W. Chen S. (2012). Whole-genome sequence of Nocardiopsis alba strain ATCC BAA-2165, associated with honeybees. *J. Bacteriol.* 194 6358–6359. 10.1128/JB.01522-12 23105086 PMC3486387

[B114] Rather S. A. Kumar S. Rah B. Arif M. Ali A. Qazi P. (2013). A potent cytotoxic metabolite from terrestrial actinomycete, Streptomyces collinus. *Med. Chem. Res.* 23 382–387. 10.1007/s00044-013-0640-2

[B115] Rodríguez V. Martín J. Sarmiento-Vizcaíno A. de la Cruz M. García L. A. Blanco G. (2018). Anthracimycin B, a Potent Antibiotic against Gram-Positive Bacteria Isolated from Cultures of the Deep-Sea Actinomycete Streptomyces cyaneofuscatus M-169. *Mar. Drugs* 16:406. 10.3390/md16110406 30366404 PMC6267485

[B116] Russell D. W. Sambrook J. F. (2001). *Molecular Cloning: A Laboratory Manual*, 3rd Edn. New York, NY: Cold Spring Harbor Laboratory Press.

[B117] Sánchez-Suárez J. Coy-Barrera E. Villamil L. Díaz L. (2020). *Streptomyces*-Derived Metabolites with Potential Photoprotective Properties-A Systematic Literature Review and Meta-Analysis on the Reported Chemodiversity. *Molecules* 25:3221. 10.3390/molecules25143221 32679651 PMC7397340

[B118] Santos O. C. S. Soares A. R. Machado F. L. S. Romanos M. T. V. Muricy G. Giambiagi-deMarval M. (2015). Investigation of biotechnological potential of sponge-associated bacteria collected in Brazilian coast. *Lett. Appl. Microbiol.* 60 140–147. 10.1111/lam.12347 25355062

[B119] Santos J. D. Vitorino I. de la Cruz M. Díaz C. Cautain B. Annang F. (2020). Diketopiperazines and other bioactive compounds from bacterial symbionts of marine sponges. *Antonie Van Leeuwenhoek* 113, 875–887. 10.1007/s10482-020-01398-2 32130598

[B120] Sarmiento-Vizcaíno A. Braña A. F. González V. Nava H. Molina A. Llera E. (2016). Atmospheric Dispersal of Bioactive *Streptomyces albidoflavus* Strains Among Terrestrial and Marine Environments. *Microb. Ecol.* 71 375–386. 10.1007/s00248-015-0654-z 26224165

[B121] Sarmiento-Vizcaíno A. González V. Braña A. F. Palacios J. J. Otero L. Fernández J. (2017b). Pharmacological Potential of Phylogenetically Diverse Actinobacteria Isolated from Deep-Sea Coral Ecosystems of the Submarine Avilés Canyon in the Cantabrian Sea. *Microb. Ecol.* 73 338–352. 10.1007/s00248-016-0845-2 27614749

[B122] Sarmiento-Vizcaíno A. Braña A. F. Pérez-Victoria I. Martín J. de Pedro N. Cruz M. (2017a). Paulomycin G, a New Natural Product with Cytotoxic Activity against Tumor Cell Lines Produced by Deep-Sea Sediment Derived *Micromonospora matsumotoense* M-412 from the Avilés Canyon in the Cantabrian Sea. *Mar. Drugs* 15:271. 10.3390/md15090271 28846627 PMC5618410

[B123] Sarmiento-Vizcaíno A. Espadas J. Martín J. Braña A. F. Reyes F. García L. A. (2018). Atmospheric Precipitations, Hailstone and Rainwater, as a Novel Source of *Streptomyces* Producing Bioactive Natural Products. *Front. Microbiol.* 9:773. 10.3389/fmicb.2018.00773 29740412 PMC5924784

[B124] Sarmiento-Vizcaíno A. González V. Braña A. F. Molina A. Acuña J. L. García L. A. (2015). *Myceligenerans cantabricum* sp. nov., a barotolerant actinobacterium isolated from a deep cold-water coral. *Int. J. Syst. Evol. Microbiol.* 65(Pt 4), 1328–1334. 10.1099/ijs.0.000107 25667397

[B125] Savic M. Bratic I. Vasiljevic B. (2007). Streptomyces durmitorensis sp. nov., a producer of an FK506-like immunosuppressant. *Int. J. Syst. Evol. Microbiol.* 57(Pt 9), 2119–2124. 17766883 10.1099/ijs.0.64913-0

[B126] Schrey S. D. Erkenbrack E. Früh E. Fengler S. Hommel K. Horlacher N. (2012). Production of fungal and bacterial growth modulating secondary metabolites is widespread among mycorrhiza-associated streptomycetes. *BMC Microbiol.* 12:164. 10.1186/1471-2180-12-164 22852578 PMC3487804

[B127] Schleissner C. Pérez M. Losada A. Rodríguez P. Crespo C. Zúñiga P. (2011). Antitumor actinopyranones produced by Streptomyces albus POR04-15-053 isolated from a marine sediment. *J. Nat. Prod.* 74, 1590–1596. 10.1021/np200196j 21718029

[B128] Schumacher R. W. Harrigan B. L. Davidson B. S. (2001). Kahakamides A and B, new neosidomycin metabolites from a marine-derived actinomycete. *Tetrahedron Lett.* 42 5133–5135.

[B129] Secor P. R. Jennings L. K. James G. A. Kirker K. R. Pulcini E. D. McInnerney K. (2012). Phevalin (aureusimine B) production by Staphylococcus aureus biofilm and impacts on human keratinocyte gene expression. *PLoS One* 7:e40973. 10.1371/journal.pone.0040973 22808288 PMC3396627

[B130] Sehgal S. N. Czerkawski H. Kudelski A. Pandev K. Saucier R. Vézina C. (1983). Ravidomycin (AY-25,545), a new antitumor antibiotic. *J. Antibiot.* 36 355–361. 10.7164/antibiotics.36.355 6853365

[B131] Shigemori H. Myung A. B. Kazunaga Y. Takuma S. Junichi K. (1992). Alteramide A, a new tetracyclic alkaloid from a bacterium Alteromonas sp. associated with the marine sponge Halichondria okadai. *J. Organic Chem.* 57 4317–4320. 10.1021/jo00041a053

[B132] Shimizu S. Suzuki M. Tomoda A. Arai S. Taguchi H. Hanawa T. (2004). Phenoxazine compounds produced by the reactions with bovine hemoglobin show antimicrobial activity against non-tuberculosis mycobacteria. Tohoku J. *Exp. Med.* 203 47–52. 10.1620/tjem.203.47 15185971

[B133] Shirling E. B. Gottlieb D. (1968). Cooperative description of type cultures of Streptomyces. II. Species descriptions from first study. *Int. J. Syst. Bacteriol.* 18 69–189.

[B134] Siegel M. R. Sisler H. D. Johnson F. (1966). Relationship of structure to fungitoxicity of cycloheximide and related glutarimide derivatives. *Biochem. Pharmacol.* 15 1213–1223. 10.1016/0006-2952(66)90286-3 5973163

[B135] Silva L. J. Crevelin E. J. Souza W. R. Moraes L. A. Melo I. S. Zucchi T. D. (2014). Streptomyces araujoniae Produces a Multiantibiotic Complex with Ionophoric Properties to Control Botrytis cinerea. *Phytopathology* 104 1298–1305. 10.1094/PHYTO-11-13-0327-R 24983843

[B136] Singh L. S. Sharma H. Talukdar N. C. (2014). Production of potent antimicrobial agent by actinomycete, Streptomyces sannanensis strain SU118 isolated from phoomdi in Loktak Lake of Manipur, India. *BMC Microbiol.* 14:278. 10.1186/s12866-014-0278-3 25406714 PMC4243295

[B137] Songia S. Mortellaro A. Taverna S. Fornasiero C. Scheiber E. A. Erba E. (1997). Characterization of the new immunosuppressive drug undecylprodigiosin in human lymphocytes: retinoblastoma protein, cyclin-dependent kinase-2, and cyclin-dependent kinase-4 as molecular targets. *J. Immunol. Baltim.* 158 3987–3995. 9103470

[B138] Stadler M. Bauch F. Henkel T. Mühlbauer A. Müller H. Spaltmann F. (2001). Antifungal actinomycete metabolites discovered in a differential cell-based screening using a recombinant TOPO1 deletion mutant strain. *Arch. Pharm.* 334 143–147. 10.1002/1521-4184(200105)334:5<143::aid-ardp143>3.0.co;2-b 11413818

[B139] Stankovic N. Senerovic L. Ilic-Tomic T. Vasiljevic B. Nikodinovic-Runic J. (2014). Properties and applications of undecylprodigiosin and other bacterial prodigiosins. *Appl. Microbiol. Biotechnol.* 98 3841–3858. 24562326 10.1007/s00253-014-5590-1

[B140] Stein A. F. Draxler R. R. Rolph G. D. Stunder B. J. B. Cohen M. D. Ngan F. (2015). NOAA’s HYSPLIT Atmospheric Transport and Dispersion Modeling System. *Bull. Am. Meteorol. Soc.* 96 2059–2077. 10.1175/BAMS-D-14-00110.1

[B141] Sullia S. B. Griffin D. H. (1977). Inhibition of DNA synthesis by cycloheximide and blasticidin-S is independent of their effect on protein synthesis. *Biochim. Biophys. Acta* 475 14–22. 10.1016/0005-2787(77)90334-3 849443

[B142] Tadtong S. Meksuriyen D. Tanasupawat S. Isobe M. Suwanborirux K. (2007). Geldanamycin derivatives and neuroprotective effect on cultured P19-derived neurons. Bioorg. *Med. Chem. Lett.* 17 2939–2943. 10.1016/j.bmcl.2006.12.041 17442565

[B143] Taechowisan T. Wanbanjob A. Tuntiwachwuttikul P. Liu J. (2010). Anti-inflammatory effects of lansai C and D cause inhibition of STAT-1 and NF-κB activations in LPS-induced RAW 264. *7 Cells Food Agricult. Immunol.* 21 57–64.

[B144] Tan X. Y. Wang X. Liu Q. S. Xie X. Q. Li Y. Li B. Q. (2018). Inhibition of silkworm vacuolar-type ATPase activity by its inhibitor Bafilomycin A1 induces caspase-dependent apoptosis in an embryonic cell line of silkworm. *Arch. Insect Biochem. Physiol.* 99:e21507. 10.1002/arch.21507 30246413

[B145] Tang Y. Q. Sattler I. Thiericke R. Grabley S. Feng X. Z. (2000). Feigrisolides A, B, C and D, new lactones with antibacterial activities from Streptomyces griseus. *J. Antibiot.* 53 934–943. 10.7164/antibiotics.53.934 11099227

[B146] Tanouchi Y. Shichi H. (1987). Immunosuppressive effects of polynactins (tetranactin, trinactin and dinactin) on experimental autoimmune uveoretinitis in rats. *Jpn. J. Ophthalmol.* 31 218–229. 3499534

[B147] Tanouchi Y. Shichi H. (1988). Immunosuppressive and anti-proliferative effects of a macrotetrolide antibiotic, tetranactin. *Immunology* 63 471–475. 3258281 PMC1454748

[B148] Thongchai T. Asawin W. Pittaya T. Jikai L. (2010). Anti-inflammatory effects of lansai C and D cause inhibition of STAT-1 and NF-κB activations in LPS-induced RAW 264. *7 Cells Food Agricult. Immunol.* 21 57–64. 10.1080/09540100903419592

[B149] Ubukata M. Osada H. Kudo T. Isono K. (1993). Respinomycins A1, A2 B, C and D, a novel group of anthracycline antibiotics. I. Taxonomy, fermentation, isolation and biological activities. *J. Antibiot.* 46 936–941. 10.7164/antibiotics.46.936 8344875

[B150] Umland S. P. Shah H. Jakway J. P. Shortall J. Razac S. Garlisi C. G. (1999). Effects of cyclosporin A and dinactin on T-cell proliferation, interleukin-5 production, and murine pulmonary inflammation. *Am. J. Respir. Cell Mol. Biol.* 20 481–492. 10.1165/ajrcmb.20.3.3266 10030847

[B151] Vaden R. M. Oswald N. W. Potts M. B. MacMillan J. B. White M. A. (2017). FUSION-Guided Hypothesis Development Leads to the Identification of N*6*,N*6*-Dimethyladenosine, a Marine-Derived AKT Pathway Inhibitor. *Mar. Drugs* 15:75. 10.3390/md15030075 28294973 PMC5367032

[B152] Vijaya Kumar E. K. Kenia J. Mukhopadhyay T. Nadkarni S. R. (1999). Methylsulfomycin I, a new cyclic peptide antibiotic from a Streptomyces sp. HIL Y-9420704. *J. Nat. Prod.* 62 1562–1564. 10.1021/np990088y 10579874

[B153] Vivian D. (1956). The Practical Synthesis of 1-Phenazinol. *Nature* 178:753. 10.1038/178753a0 13369535

[B154] Vu H. T. Nguyen D. T. Nguyen H. Q. Chu H. H. Chu S. K. Chau M. V. (2018). Antimicrobial and Cytotoxic Properties of Bioactive Metabolites Produced by Streptomyces cavourensis YBQ59 Isolated from Cinnamomum cassia Prels in Yen Bai Province of Vietnam. *Curr. Microbiol.* 75 1247–1255. 10.1007/s00284-018-1517-x 29869093

[B155] Wang D. Wang C. Gui P. Liu H. Khalaf S. M. H. Elsayed E. A. (2017). Identification, Bioactivity, and Productivity of Actinomycins from the Marine-Derived *Streptomyces heliomycini*. *Front. Microbiol.* 8:1147. 10.3389/fmicb.2017.01147 28702007 PMC5487404

[B156] Wang X. M. Wang X. Dong M. Li Z. M. Liu Z. X. (2020). Synthesis and biological activities of 1H-indole-1-carboxylic acid aryl esters as a marine antifouling coating. *J. Coat Technol. Res.* 17 553–561

[B157] Welander Paula V. Ryan C. H. Lichun Z. Alex L. S. Roger E. S. Dianne K. N. (2009). Hopanoids Play a Role in Membrane Integrity and pH Homeostasis in *Rhodopseudomonas palustris* TIE-1. *J. Bacteriol.* 191 6145–6156. 10.1128/JB.00460-09 19592593 PMC2747905

[B158] Williamson N. R. Fineran P. C. Leeper F. J. Salmond G. P. C. (2006). The biosynthesis and regulation of bacterial prodiginines. *Nat. Rev. Microbiol.* 4 887–899. 10.1038/nrmicro1531 17109029

[B159] Wu C. Z. Jang J. H. Ahn J. S. Hong Y. S. (2012). New geldanamycin analogs from Streptomyces hygroscopicus. *J. Microbiol. Biotechnol.* 22 1478–1481. 10.4014/jmb.1206.06026 23124337

[B160] Xu J. Yang Q. (2010). Isolation and characterization of rice straw degrading Streptomyces griseorubens C-5. *Biodegradation* 21 107–116. 10.1007/s10532-009-9285-8 19597946

[B161] Ye L. Zhang H. Xu H. Zou Q. Cheng C. Dong D. (2010). Phenazine-1-carboxylic acid derivatives: design, synthesis and biological evaluation against Rhizoctonia solani Kuhn. *Bioorg. Med. Chem. Lett.* 20 7369–7371. 10.1016/j.bmcl.2010.10.050 21055934

[B162] Zhan Y. Zheng S. (2016). Efficient production of nonactin by Streptomyces griseus subsp. griseus. *Can. J. Microbiol.* 62 711–714. 10.1139/cjm-2016-0248 27405846

