# Supplementary material for: Bioactive Natural Products in Actinobacteria Isolated in Rainwater From Storm Clouds Transported by Western Winds in Spain
Source: Front Microbiol. 2021 Nov 10;12:773095. doi: 10.3389/fmicb.2021.773095 (PMC8631523; doi:10.3389/fmicb.2021.773095)
Supplement: Supplementary Material 1 — UV210 nm chromatograms corresponding to all samples. [file Data_Sheet_1.zip › Supplementary Material 1.DOCX]

*Nocardiopsis* sp. A-45

*Streptomyces* sp. A-50

*Streptomyces* sp. A-53

*Streptomyces* sp. A-69

*Streptomyces* sp. A-87

*Streptomyces* sp. A-139

*Streptomyces* sp. A-167

*Nocardiopsis* sp.A-169

*Streptomyces* sp. A-171

*Streptomyces* sp. A-178

*Streptomyces* sp. A-179

*Streptomyces* sp. A-241

*Streptomyces* sp. A-249

*Streptomyces* sp. A-250

*Streptomyces* sp. A-254

*Nocardiopsis* sp. A-256

*Nocardiopsis* sp. A-257

*Streptomyces* sp. A-257

*Nocardiopsis* sp. A-260

*Streptomyces* sp. A-261

*Streptomyces* sp. A-262

*Streptomyces* sp. A-263

*Streptomyces* sp. A-265

*Streptomyces* sp. A-266

*Streptomyces* sp. A-268

*Streptomyces* sp. A-269

*Streptomyces* sp. A-271
