# Supplementary material for: Bioactive Natural Products in Actinobacteria Isolated in Rainwater From Storm Clouds Transported by Western Winds in Spain
Source: Front Microbiol. 2021 Nov 10;12:773095. doi: 10.3389/fmicb.2021.773095 (PMC8631523; doi:10.3389/fmicb.2021.773095)
Supplement: Supplementary Material 1 — UV210 nm chromatograms corresponding to all samples. [file Data_Sheet_1.zip › Supplementary Material 2.DOCX]

| **Producer strain** | **Molecular formula of compounds unmatched**  **in DPN** | **Comment** |
| --- | --- | --- |
| *Streptomyces* sp. A-53 | C_24_H_23_NO_6_S  C_28_H_22_O_6_ |  |
| *Streptomyces* sp. A-87 | C_44_H_71_N_9_O_9_ |  |
| *Streptomyces* sp. A-139 | C_18_H_27_NO_10_  C_49_H_57_ClO_12_*  C_49_H_57_ClO_11_ |  |
| *Streptomyces* sp.A-167 | C_9_H_7_NO_4_  C_28_H_39_NO_3_ |  |
| *Nocardiopsis* sp. A-169 | C_35_H_68_N_4_O_16_  C_29_H_53_NO_11_ |  |
| *Streptomyces* sp.A-171 | C_15_H_11_NO_4_ |  |
| *Streptomyces* sp.-249 | C_14_H_26_O_5_ |  |
| *Streptomyces* sp.A-254 | C_28_H_31_NO_8_  C_30_H_33_NO_9_  C_40_H_57_NO_10_ |  |
| *Streptomyces* sp.A-258 | C_28_H_37_N_3_O_6_  C_28_H_33_N_3_O_6_  C_21_H_40_N_2_O_4_ | Related to virginiamycin |
| *Streptomyces* sp. A-261 | C_21_H_17_N_2_O_2_  C_19_H_21_NO_5_  C_26_H_34_N_2_O_5_  C_12_H_20_O_2_S |  |
| *Streptomyces* sp.A-262 | C_22_H_16_O_9_  C_23_H_18_O_9_ |  |
| *Streptomyces* sp. A-266 | C_23_H_32_N_2_O_9_ |  |
| *Streptomyces* sp. A-269 | C_18_H_30_O_4_* | Related to albocycline |
| *Streptomyces* sp*.* A-271 | C_9_H_15_NO_2_  C_17_H_29_NO_6_ |  |

* Detected at two different retention times
